# Investigating potassium silicate efficacy and mechanisms for improving the strawberry agronomic traits and gray mold fungal resistance

**DOI:** 10.7717/peerj.21151

**Published:** 2026-04-29

**Authors:** Amaranatha R. Vennapusa, Rosalyn Battle, Surya Krishna Sakthivel, Venkata H. Limmada, Michael Moore, Jayesh Samtani, Sathya Elavarthi, Kalpalatha Melmaiee

**Affiliations:** 1 Department of Agriculture and Natural Resources, Delaware State University, Dover, DE, United States; 2 Optical Science Center for Applied Research (OSCAR), Delaware State University, Dover, DE, United States; 3 Hampton Roads Agricultural Research and Extension Center, School of Plant and Environmental Sciences, Virginia Polytechnic Institute and State University (Virginia Tech), Virginia Beach, VA, United States

**Keywords:** Strawberry, Potassium silicate, Gray mold, Fruits, Yield, Phytoliths

## Abstract

Strawberries are a high-value crop in the United States due to their increasing demand and nutritional benefits. Strawberry production faces significant losses due to gray mold fruit rot caused by *Botrytis cinerea*. While fungicides provide major control, concerns about residues and the evolution of fungicide resistance demand alternative approaches to disease management. In this context, our study evaluated the potential use of potassium silicate (K_2_SiO_3_) to enhance agronomic traits in plants, elucidate its role in combating gray mold in strawberries, and identify the responsible genes. Two strawberry cultivars (Flavorfest and Rutgers Scarlet TM) grown under high-tunnel field conditions were foliar-sprayed with different doses of potassium silicate (K_2_SiO_3_) (0, 2, 3, and 4 mL per gallon of water). Silicon accumulation and agronomic traits were measured, and antifungal effects were assessed through *in vitro* assay and post-harvest fruit treatments using Chandler and Ruby June cultivars and molecular analysis using the silicon transporter genes. The 2 mL per gallon treatment showed the highest silicon concentration in leaves and phytolith formation, with limited translocation to roots, and significantly increased plant width and marketable yield. The effective concentration (2 mL/gallon) also reduced B. *cinerea* growth under *in vitro* conditions and lowered infection on postharvest-treated fruits, coinciding with enhanced expression of silicon transporter genes. Our study reports that moderate application of K_2_SiO_3_ as a foliar spray improved plant growth and production, suppressed gray mold under *in vitro* conditions, and in postharvest treatments. Increased expression of transporter genes indicates the plant's response to its application.

## Introduction

The strawberry (*Fragaria × ananassa* Duch.) is one of the most widely adored berries internationally, valued for its exceptional taste, nutritional richness, and health benefits (Hassan et al., 2021; Madhavi et al., 2023; Yeh et al., 2023). In 2023, the United States ranked second in global production with 1.38 million tons of fruit. The strawberry market has steadily grown over the past decade and is predicted to sustain this trend in the coming years. Within the United States, California is the top producer of strawberries (91%), followed by Florida (8%), which is the major producer for the winter market (Dong et al., 2020; Hernández-Martínez et al., 2023). Based on strawberry production, the United States is divided into eight geographic regions (Samtani et al., 2019). Region 6 encompasses the Northeast and Mid-Atlantic states, including Vermont, Rhode Island, Connecticut, Maine, New Hampshire, New York, New Jersey, Delaware, Pennsylvania, Maryland, and West Virginia. This production region is significant for local produce and small marketing industries. However, several challenges are faced in this region in producing strawberry crops, including adverse environmental conditions, pest and disease infestations, and insufficient labor during the harvest season. These challenges are similar to those encountered in other regions as well (Manzoor et al., 2023; Samtani et al., 2019). Strawberry plants are vulnerable to harsh environmental conditions, and therefore, new strategies are needed to strengthen plant health, agronomic traits, and yield (Gulen, Turhan & Erİs, 2016).

*B. cinerea* is one of the major fungal pathogens that causes a devastating gray mold disease in strawberries by infecting various plant organs at different developmental stages in the field with 50–80% yield loss (Bhaskara Reddy et al., 2000; Hassan et al., 2021; Lewers, Luo & Vinyard, 2012; Zhang et al., 2023). The infection begins on senescent flowers while they are still attached to the plant, then spreads to adjacent developing fruit. Gray mold symptoms appear as soft rot with water-soaked parenchyma, followed by masses of gray conidia on leaves and flowers. The fungus persists on crop debris as mycelia and survives as conidia and sclerotia during winter, providing a source of inoculum for the next crop generation (Williamson et al., 2007). Hence, it is difficult to control due to its many modes of attack and various inoculum sources. Consequently, *B. cinerea* infection in strawberry production causes severe economic losses during preharvest growth, handling processes, and postharvest storage.

Gray mold management in strawberries relies heavily on repeated applications of chemical fungicides, including quinone outside inhibitors (QoI: azoxystrobin) and benzimidazoles (thiabendazole), as well as multisite fungicides such as captan. Although these products are effective, they are largely preventive rather than curative and require frequent use, which led to the emergence of fungicide-resistant *B. cinerea* populations, especially against QoI fungicides, limiting their long-term efficacy. At the same time, economic and environmental concerns, including pesticide residues and human health risk, phytotoxicity, soil and water contamination, are demanding safer and sustainable approaches for sustainable disease management (Hassan et al., 2021; Liu, Davies & Hall, 2020; Williamson et al., 2007).

Several chemical alternatives have been explored, including biological agents such as bacteria, yeast, and filamentous fungi, including Trichoderma, Gliocladium, Rhodotorula, and Metschnikowia (Di Francesco et al., 2024; Elad & Stewart, 2007; Freimoser et al., 2019). Other approaches include biofumigation using natural volatile compounds, plant extracts, essential oils, and plant hormones for both pre- and post-harvest treatments (Abubakar, Koul & Sharma, 2025; Gulzar et al., 2025a, 2025b; Nazir et al., 2015; Soppelsa et al., 2024). Even though the development of resistant cultivars offers a long-term solution, progress is slow due to complex inheritance patterns and genotype × environment interactions. Consequently, mineral-based supplements that enhance plant defense responses have gained interest, with silicon (Si) emerging as a particularly promising, eco-friendly candidate for reducing disease severity.

Si is the second most abundant element in the Earth’s crust. Si is absorbed by plants in the silicic acid (H_4_SiO_4_) form, which is present in the soil solution (Etesami & Jeong, 2018). Si has been recognized as a nutrient for plants by the International Plant Nutrition Institute (IPNI) (http://www.ipni.net/nutrifacts-northamerican). Furthermore, Si has been officially declared by the Association of American Plant Food Control Officials (AAPFCO) as a plant’s “beneficial substance” (Zargar et al., 2019). Si has been reported to be beneficial for plant growth and development, and its accumulation and deposition form a double silicate layer in the cuticle of leaves, which helps reduce evapotranspiration and improve the photosynthetic rate (Park et al., 2018; Wang et al., 2017). Additionally, the Si deposition-induced variations in plants can reinforce the morphological structure of the cell wall and maintain the cell membrane integrity by creating a physical barrier (Ma & Yamaji, 2015; Nasircilar & Ulukap, 2021; Zargar et al., 2019).

Si treatments in strawberries and other crop plants have proven to ameliorate the consequences of abiotic and biotic stresses (Abd-El-Kareem, Elshahawy & Abd-Elgawad, 2019; Dehghanipoodeh et al., 2018; Wang & Galletta, 1998; Wang et al., 2017; Xiao, Li & Jeong, 2022; Yaghubi et al., 2016; Zargar et al., 2019). Previous research has demonstrated the efficacy of Si in reducing different fungal diseases in strawberries (Abd-El-Kareem, Elshahawy & Abd-Elgawad, 2019; Liu, Davies & Hall, 2020; Ouellette et al., 2017). However, very limited information exists regarding their response to *B*. *cinerea*, and understanding Si accumulation in plants, disease resistance, and mechanisms (Lopes et al., 2014; Romero et al., 2022). Therefore, there is a critical need for further investigation into Si treatments and their role in improving plant health and resistance to fungal diseases in strawberries, as an alternative to chemical fungicides.

Si foliar sprays are commonly used to prevent fungal diseases in various crops and plants, including strawberries, capsicum, date palms, mangoes, grapes, papayas, peppers, potatoes, rice, and sugar cane, and increase fruit size and yield in some of these crops (Laane, 2018; Petrasch et al., 2019; Zargar et al., 2019). While earlier studies have suggested that K_2_SiO_3_ applications benefit strawberry crop production by improving plant health and yield upon pot cultures and edible Si fruit treatments (Jalal et al., 2024; Jarosz et al., 2025; Wang et al., 2017). But there is no systematic research to determine the most effective dose, assess improvements in agronomic traits under high-tunnel field conditions, confirm Si absorption and accumulation rates in plants, and identify the resistance mechanisms involved against *B. cinerea*. Based on this, our study hypothesizes that optimizing the effective dose, enhancing si accumulation, and elucidating its preventive role against *B. cinerea* provide chemical-fungicide alternatives to improve strawberry production and disease management. Furthermore, our specific objectives are to evaluate how K_2_SiO_3_ affects strawberry plant growth and agronomic traits under high-tunnel field conditions and to assess Si accumulation in plant tissues using microscopy. Additionally, the efficacy of K_2_SiO_3_ against *B. cinerea* was assessed using an *in vitro* fungal assay and postharvest fruit treatments, and the molecular mechanisms underlying resistance were elucidated through a gene-expression study. Portions of the introduction were previously published as a preprint (Vennapusa et al., 2024).

## Materials and Methods

### Plant propagation

‘Flavorfest’ and ‘Rutgers Scarlet™’ strawberries were purchased as bare roots (Nourse Farms, South Deerfield, MA, USA) and planted on March 27, 2018, inside a high tunnel at the Delaware State University Outreach and Research Center, Smyrna, Delaware (DE), USA. The soil (sandy loam) was amended with a compost mixture of 50% spent mushroom and 50% poultry manure. Before planting and at the end of the study, soil plugs were collected 2.1M apart in a “Z” formation inside the high tunnel and submitted to the University of Delaware Soil Testing Paradee Center, Dover, DE, USA, for soil analysis. The soil report is given in Figs. S1 & S2. Bare roots were planted in a split-block design consisting of nine plots with a size of 9 m × 1 m plot. In each plot, 26 strawberry bare root plants were planted, and once the plants were established, they were reduced to a sample size of 12 per plot with a spacing of 30–45 cm between plants. Out of nine plots, three were planted with the ‘Flavorfest’ cultivar, three with the ‘Rutgers Scarlet™’ cultivar as treatment plots, and the remaining three plots served as controls with six plants of each cultivar. Uniform irrigation was provided to both control and silicon-treated plants *via* drip irrigation under natural sunlight and ambient temperature conditions.

### Foliar spray of Si treatment

The plots were randomly assigned for the treatments as: T₀: untreated (water), T_1_: 2 mL, T_2_: 3 mL, and T_3_: 4 mL of potassium-silica (Gold Shield Silica Supplement (0-0-1), Blue Planet Nutrients®) per 3.78 liters (one gallon) of water. The potassium silicate doses and number of treatments (0, 2, 3, and 4 mL per gallon) were selected based on the recommendation of the Blue Planet Nutrients Gold Shield (dilute 1–2 mL per gallon for seedlings/small plants or 2–4 mL for medium/large-also great as foliar spray 1 mL per quart water, weekly). The Si treatments were foliar sprayed in five Si applications in the fall of 2018 and spring of 2019 using a Shindaiwa (model SP53) manual backpack sprayer. The 28-inch wand was held approximately two inches above the plant canopy for 12 s, applying 90 psi of potassium silica solution onto each cultivar’s leaf adaxial and abaxial surfaces.

### Sample preparation for scanning electron microscopy with energy dispersive X-ray spectroscopy (SEM-EDS) and spectrophotometer analysis

The dry ash method was followed to extract silicon particles known as phytoliths from the plant material. Fresh strawberry leaf material from the control and treatment plants was collected and air-dried for 3 days. Ten grams of dry material were crushed using a pestle and transferred to the sterile 50 mL polypropylene conical tubes with distilled water (DW). The tubes were then placed in an ultrasonic bath (Model 1510; Ultrasonic Cleaner, Branson) for 15 min to remove particulate matter from the surface and then transferred into a crucible to dry at 65 °C for 24 h (h) in a dry oven (Model 10 Lab Oven; Quincy Lab Inc., Burr ridge, Illinois, US). The crucible was placed in a muffle furnace (Isotemp® Muffle Furnace, Fisher Scientific, Waltham, MA, USA) for 6 h at 500 °C; afterward, it was moved into a desiccator to cool. Then, the sample was transferred to a beaker and soaked in 10% HCl for 30 min to remove carbonates and triple-rinsed in DW. Under a fume hood, a ratio of 2:1, 65% HNO_3_ (nitric acid) and 70% HClO_4_ (perchloric acid), respectively, was added to the sample and placed in a sand bath apparatus at 80 °C for 16 h. After digestion, hydrogen peroxide was added to the sample, kept in a sand bath for 16 h at 65 °C, and then rinsed with DW three times (Snyder, 2001). Two sets of samples were made for the experiments. One group of samples was used for spectrophotometer quantification, and the other was used for SEM-EDS analysis at the Imaging facility OSCAR Building, Delaware State University (DSU).

### Specimen preparation for SEM-EDS image analysis

The leaf and root samples were adhered to aluminum stubs for observation under Scanning Electron Microscopy (SEM) (Quanta 250-FEG; FEI Hillsboro, OR, USA) associated with Energy-Dispersive X-Ray Spectroscopy (EDS) (X-Man, Oxford, Cambridge, MA, USA). To the sample, 500 mL of DW was added and vortexed, then 20 µL was aliquated into test tubes containing 1 mL of nanopore DW. The test tubes containing the sample were vortexed, and then 150 μL were pipetted onto the top of the carbon tape adhesive stubs and allowed to dry for 24 h. Once the samples were dry, the stubs were coated with gold using a Quorum 150R ES plus. EDS mapping of the entire field of view was performed at each sample location. EDS spectra of Si, K, and Au were collected from the organic materials and carbon tape and analyzed with Aztec software (Oxford Instruments, Cambridge, MA, USA). SEM uses excited electrons for images, and the EDS tool measures the energy of emitted photons in the X-ray electromagnetic spectrum to quantify the elemental composition of a sample. Micron EDS map images were created for each leaf and root sample to determine phytolith weight percentages (Abed-Ashtiani et al., 2012).

### Silicomolybdic acid assay using the spectrophotometric method

The silica concentrations in leaf and root samples were estimated using a spectrophotometric silicomolybdate technique (Allen, 1989; Manning, Routoula & Patwardhan, 2018; Massey et al., 2008). The Molybdenum Blue Reagent (MBR) stock solution was prepared by adding 10 g (8 mmol) of ammonium molybdate tetrahydrate to 500 mL of DW and subjecting it to gentle stirring. The solution was acidified by carefully adding 60 mL of 10 M HCl solution, and the final volume was made to 1 L. The para-aminophenol sulfate reducing reagent was prepared by dissolving 19.5 mmol (3.35 g) of para-aminophenol sulfate, 111 mmol (10 g) of anhydrous oxalic acid, and 16 mmol (2 g) of sodium sulfite in 250 mL of DW and by slowly adding 92 g (50 mL) of saturated sulfuric acid while stirring and final volume was made up to 500 mL with DW. The blank (DW) and plant samples (10 μL) were diluted in MBR (300 µL of MBR 3 mL of DW) solution and shaken well (this solution will slowly turn yellow). After that, 1.6 mL of the reducing reagent was added to reduce the yellow silicomolybdate complex to its blue isomer. Later, the samples were allowed to develop a quantifiable blue color for at least 2 h, and absorbance was measured at 810 nm using a UV-vis spectrophotometer. The Si concentrations were calculated based on the linear relationship between absorbance and concentration (linear calibration: R^2^ = 92%).

### Plant canopy and fruit yield

Plant height and area were recorded with a measuring tape for each plant. Strawberries were harvested in spring 2019, two to three times a week as needed, and sorted as marketable and non-marketable fruit based on USDA standards, in which strawberries are classified by size and defects (Flanagan et al., 2020). During harvest, fruit was separated into marketable and nonmarketable categories. The marketable fruit comprised fruit that was 90% to 100% red in color on the external surface with calyx attached, and free of mold, diseases, insect, bird, or animal damage. The unmarketable fruit is categorized based on damage, such as unhealthy, diseased, misshapen, or deformed fruits.

### *In-vitro* evaluation of the antifungal activity of silica against *B. cinerea*

The antifungal activity of K_2_SiO_3_ against *B. cinerea* was assessed by measuring the fungal mycelial radial growth using a modified method (Mendoza et al., 2005). *B. cinerea* fungal strain was a generous gift from Dr. M. Mahfuz Rahman, Associate Professor of Plant Pathology Extension Specialist, West Virginia University, Morgantown, WV, USA. *B. cinerea* mycelial plugs were placed in the middle of the Petri dishes containing potato dextrose agar (PDA; Thermo Scientific™ Oxoid™) medium supplemented with streptomycin (300 mg/l) and different concentrations of sterile K_2_SiO_3_ solutions (1, 2, 3, 4, and 5 mL/L), and no K_2_SiO_3_ was added to control plates. After inoculation, the Petri dishes were sealed with parafilm and incubated in the growth chamber at 25 °C with 90% humidity for 5 days. Radial growth of the fungus was measured in diameters daily for 5 days (days after inoculation, DAI) using a vernier caliper, and the area was calculated (Slowik et al., 2023). Three biological replicates were maintained for each treatment.

### Postharvest treatment of strawberry fruits with potassium silicate and assessment of *B. cinerea* resistance

The efficacy of K_2_SiO_3_ treatments against *B. cinerea* resistance was assessed using the postharvest strawberry fruit assay. Fruits from two strawberry cultivars, Chandler and Ruby June, were harvested from the open field in the 2024 growing season at Hampton Roads Agriculture Research and Extension Center in Virginia Beach, VA, and shipped to DSU for fruit assay. The cultivars for this experiment were selected based on fruit availability, and to test whether the K_2_SiO_3_ treatments were effective across different genetic backgrounds as post-harvest treatments. Physiologically matured healthy strawberry fruits free from wounds were selected, rinsed with running tap water, then with Milli-Q water, and surface sterilized with 2% soap water (Liqui-Nox detergent) and ten times diluted commercial bleach (8.25% sodium hypochlorite) and fungizone (2 μg/mL Amphotericin B, protects from other fungal infections and contamination) using an in-house method with minor modifications (Limmada et al., 2026). After each treatment, fruits were rinsed three times with autoclaved Milli-Q water to remove chemical residues. The sterilized fruits were brought to Biosafety cabinets and immersed in the Tween-80 solution (control) and different concentrations of K_2_SiO_3_ (0, 1, 2, 3, and 4 mL/L) for 15 min (Nikagolla, Udugala-Ganehenege & Daundasekera, 2019). The fruits were blotted with sterilized paper towels to remove excess solutions and inoculated with uniform circular PDA plugs of (8 mm) control (no fungus) and 7-day-old *B. cinerea* actively growing mycelium in the middle of the fruits treated with different concentrations of K_2_SiO_3_. Four biological replicates for each genotype and treatment were maintained, with three technical replicates (fruits). The infected fruits were placed in sterilized containers and incubated at 10 °C and 90% humidity for 5 days in growth chambers (Adaptis A1000; Conviron, Canada). The percent injury was calculated using a 0–100% scale: 0 being no infection and 100 being highly injured fruit surface area by the fungal infection. A set of fruit samples was flash-frozen and stored for molecular studies.

### Gene expression study using quantitative real-time PCR (RT-qPCR)

RT-qPCR was performed to assess the function of the silicon transporter genes in one of the strawberry cultivars, Chandler, by postharvest fruits treated with K_2_SiO_3_ and *B. cinerea*. Total RNA from postharvest fruit samples treated with 0, 1, 2, 3, and 4 mL of K_2_SiO_3_ and *B. cinerea* was extracted using the Spectrum Plant Total RNA Kit (Sigma-Aldrich, St Louis, MO, USA) (Mulozi et al., 2023). The total RNA was estimated using the Nanodrop 2000/2000C (Thermo Scientific, Waltham, MA, USA) located in the J.W. Baker Building, Department of Agriculture and Natural Resources, Delaware State University. The gene primers used in this study were designed by Primer 3 software and custom-synthesized from IDT (Integrated DNA Technologies, Coralville, IA, USA), and sequence information was provided in Table S1. A total of 7,500 Real-Time PCR system (Applied Biosystems, Waltham, MA, USA) was used for quantitative gene expression analysis located at Ag Annex Building, Department of Agriculture and Natural Resources, Delaware State University. For each treatment, three biological and three technical replicates were maintained. The reaction mixture contained 7.5 μL of Power SYBR™ Green RNA-to-CT™ 1-Step Kit master mix (Applied Biosystems, Waltham, MA, USA), 0.15 μL of RT enzyme mix, 1.5 μL of each of the forward and reverse primers (5 μM), and 50 ng template RNA (2 μL) in a final volume of 15 μL. The thermocycler program was set at 95 °C for 5 min, followed by 35 cycles of 95 °C for 30 s and 60 °C for 30 s. Relative gene expression was normalized using *FaGADPH2* as the reference gene and relative to control (no fungal and chemical treatment) samples (Livak & Schmittgen, 2001; Vennapusa et al., 2020).

### Statistical analysis

A two-way analysis of variance (ANOVA) was used to assess differences between Si treatments and genotypes for the measured traits in the high-tunnel experiment. One-way ANOVA analysis was used for *in vitro* assay, postharvest fruit assay, and gene expression studies to identify the significant differences (Pandian et al., 2020). The bar graphs were generated using the R package ggplot2 (Pandian et al., 2023; Vennapusa et al., 2022; Wickham & Sievert, 2009). Fisher’s LSD test was used to separate the mean of significant treatments at *P* ≤ 0.05 by using the R package agricolae (de Mendiburu, 2014; Pandian et al., 2023). The alphabets in tables and graphs represent significant differences between the control and other treatments (*P* ≤ 0.05).

## Results

### Si absorption and translocation patterns among strawberry leaf and root samples

**Figure 1:**
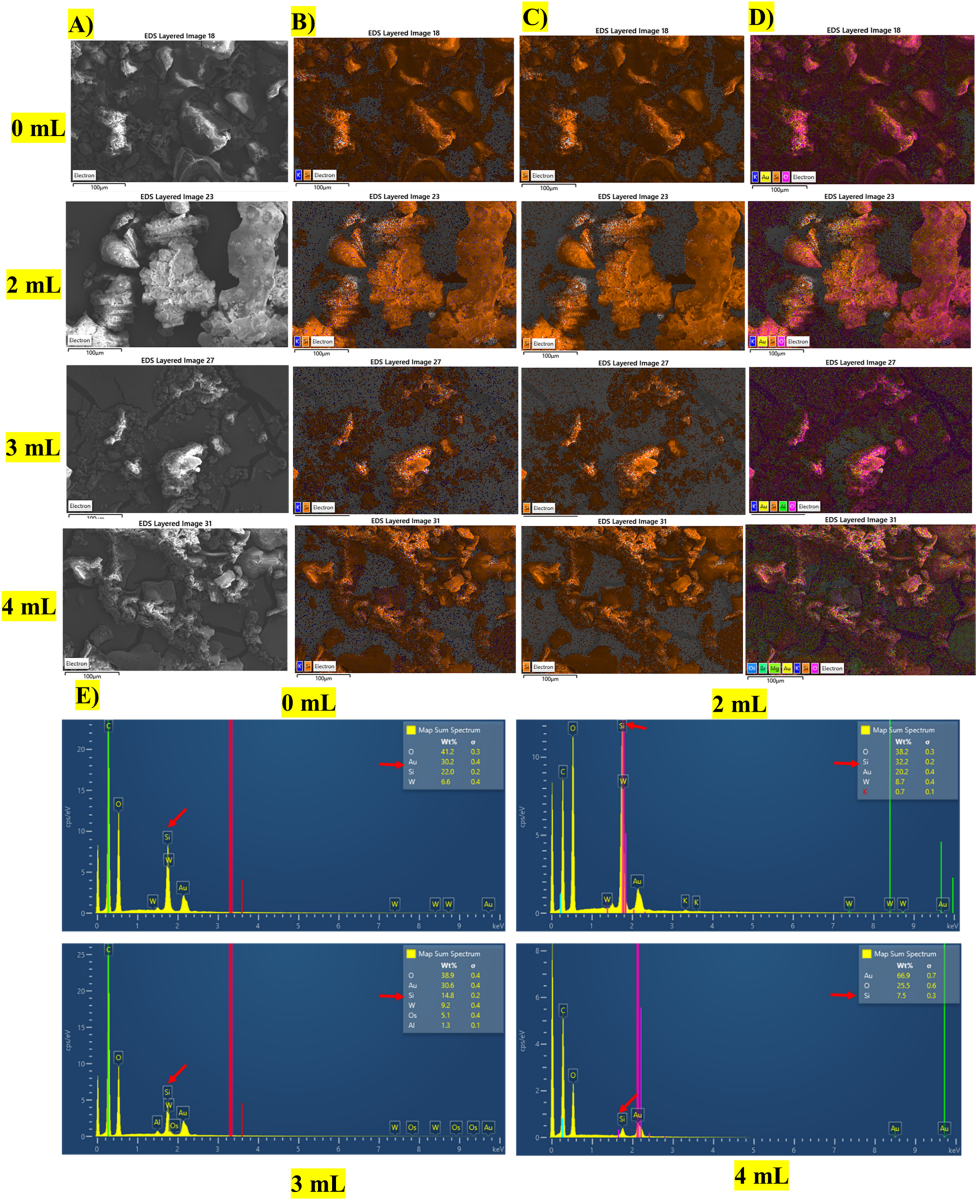
Examining the silica distribution in strawberry leaf samples using Scanning Electron Microscopy images coupled with Energy Dispersive X-ray Spectrometry (SEM-EDS). (A) In the black and white panel, white particles represents the Si accumulation and phytoliths formation (Si). (B) In this panel, orange particles represents the silica and blue for potassium (K). (C) In this panel, elemental maps represent the orange particles of silica alone. (D) Elemental maps represent the overlay of all particles, silica (Si, orange), potassium (K, blue), gold (Au, gold), and oxygen (O, pink), as indicated. Maps collected at 10 kV over the SEM image. Image width = 100 µm. (E) EDS spectrum shows the peak area of strawberry leaf samples treated with different concentrations of potassium silicate. Control (0 mL), 2 mL treatment, 3 mL treatment, and 4 mL treatment.

SEM-EDS results from the foliar-applied K_2_SiO_3_ in strawberry plant samples showed silica distribution and intensity variations. In the elemental maps, Fig. 1 panel-A of SEM image in black and white shows the “knobs” of silica in white precipitation; however, the high intensity of white precipitate in 2 mL treated plant leaf samples suggests the higher accumulation of silica. In panel B, the high-intensity orange color for the element Si indicates a higher presence of Si than potassium, represented in blue. The high intensity of orange color in 2 mL treated leaf samples suggests a higher accumulation of Si than in other samples in panels B, C, and D. (Figs. 1A–1D). EDS spectra from the strawberry leaf samples treated with 2 mL of K_2_SiO_3_ show a high peak area compared to other treatments (0, 3, and 5 mL), as well as well-structured phytoliths. Other elements, such as O and Au (internal coating material for the sample fixation), were also observed in higher intensities (Fig. 1E). On the other hand, the 2 mL treated root sample showed less intensity of orange color compared to other treatments. Higher intensity of Si accumulation was observed with increased concentrations of foliar sprays, suggesting the higher translocation of silica into the root samples from leaves with increased concentrations of foliar sprays (Figs. S3A–S3D). The EDS spectra in the root samples for 2 mL of K_2_SiO_3_ treatment showed less peak area of Si than other treatments (Fig. S3E), increasing the Si peak area observed on 3- and 5-mL treatment root samples.

**Figure 2:**
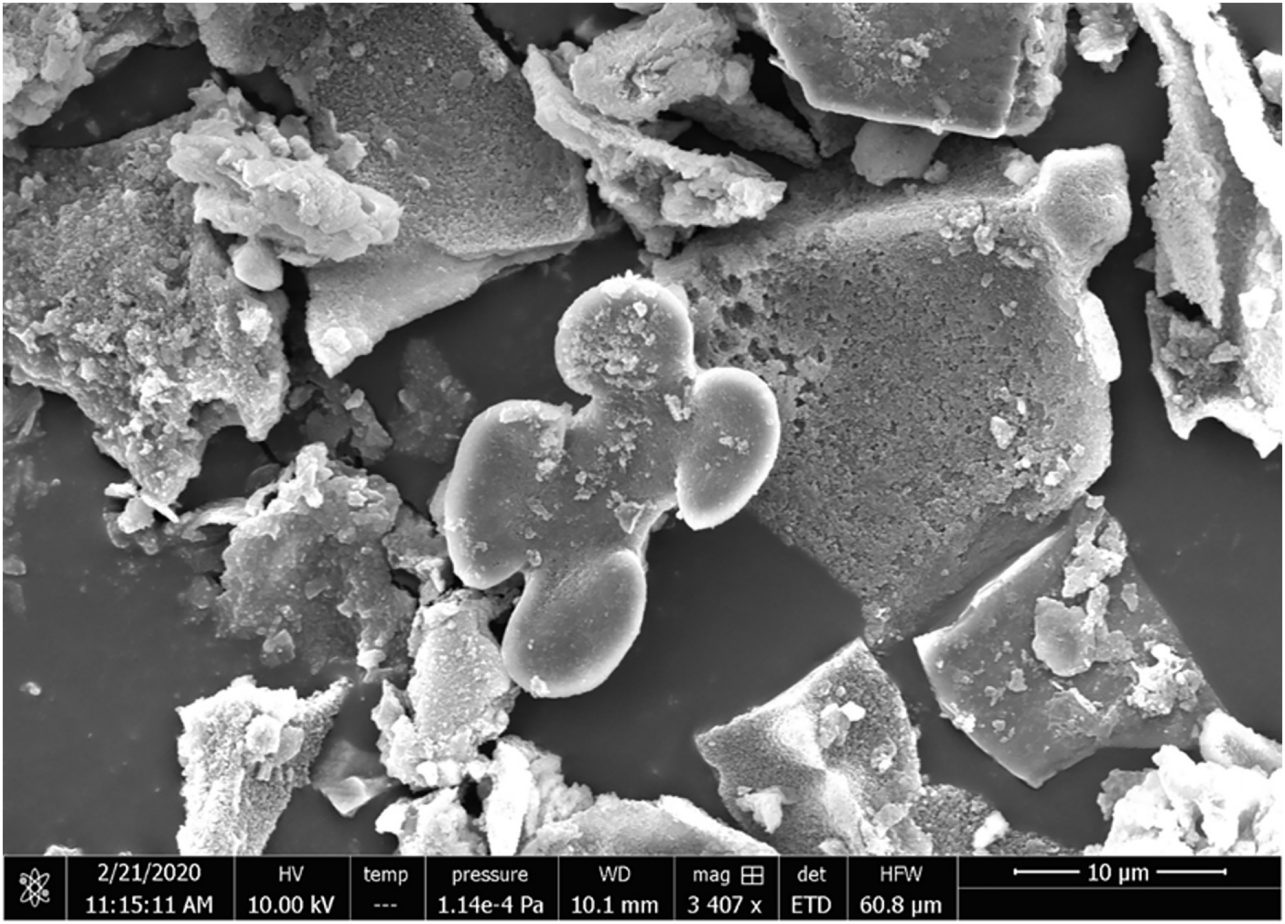
Dumbbell-shaped phytolith found in potassium silica-treated (2 mL/gallon) strawberry leaf samples.

Overall, the SEM-EDS image analysis suggests that the 2 mL treatment is effective in absorbing and retaining the Si in leaf samples compared to other treatments, and the dumbbell-shaped phytoliths of silica structures were observed in the strawberry leaves (Fig. 2).

### Si accumulation in strawberry plants

In addition to SEM-EDS data, we quantified silicon content spectrophotometrically across different strawberry plant parts to corroborate the quantitative results. The interaction between different K_2_SiO_3_ treatments was significant in strawberry leaf (*P* < 0.001) and root (*P* < 0.01) samples. Spectrophotometer analysis of leaf samples revealed a higher accumulation of silicon (48 mol/L) upon 2 mL treatments than other treatments (0 mL–23 mol/L, 3 mL–33 mol/L, and 4 mL–42 mol/L) (Fig. S4A). On the other hand, root samples accumulated a less amount of silica in 2 mL treatment (119 mol/L) compared to other treatments (0 mL–151 mol/L, 3 mL–141 mol/L, and 5 mL–226 mol/L, Fig. S4B), suggesting the 2 mL treatment shows lower translocation of silica to the roots and well partition in the leaf samples; the same pattern was visible in the 3 and 4 mL samples.

### Potassium silicate efficacy for improving the physiological and agronomic traits in strawberry plants

The strawberries grown under high tunnels were subjected to four different K_2_SiO_3_ treatments at 2-week intervals in the Fall of 2018 and Spring of 2019. Plant height, width, marketable (MKY), and non-marketable (NMY) fruit yields were recorded. The interaction between the K_2_SiO_3_ treatments and yield (marketable and non-marketable) and canopy size (width) was significant (*P* < 0.05). However, no interaction between the genotype and treatment was observed for any measured morpho-physiological traits except for the plant width (Table 1). There was no significant difference between the treatments for plant height among the two genotypes; however, plant width was significantly higher in the 2 mL treatment (183 cm) in ‘Flavorfest’ compared to other treatments (0 mL–150 cm, 3 mL–156 cm, and 4 mL–152 cm). The 2 mL/gallon potassium silicate treatment in our experiment showed the most consistent positive effects on yield and plant width among the two cultivars. On the other hand, higher concentrations (3 and 4 mL) of treatments showed reduced or nonsignificant effects on most traits. However, the 3 mL treatment resulted in a higher marketable yield for the ‘Flavorfest’ cultivar (88.33 g *vs*. 80.20 g at 2 mL), and that effect was non-significant, but total yield (marketable + non-marketable) is still high at 2 mL treatment (108.6 *vs*. 101.6 at 3 mL), also plant height, and width were greater at the 2 mL concentration. This suggests the possible broader beneficial effects of the 2 mL treatment on plant growth beyond yield alone.

**Table 1:** Efficacy of potassium silica foliar spray for improving plant growth and yield traits in strawberry plants under high tunnel conditions.

Treatment	Marketable yield (g/plant)		Non-marketable yield (g/plant)		Plant height (cm)		Plant width (cm)	
	Flavorfest	Rutgers scarlet^TM^	Flavorfest	Rutgers scarlet^TM^	Flavorfest	Rutgers scarlet^TM^	Flavorfest	Rutgers scarlet^TM^
0 mL	33.00^b^	31.57^b^	13.20^bc^	9.35^c^	20.22	19.55	150.02^b^	143.21^b^
2 mL	80.20^a^	82.80^a^	28.40^a^	24.00^ab^	22.83	19.41	183.16^a^	152.81^b^
3 mL	88.33^a^	40.00^ab^	13.60^abc^	13.60^abc^	19.91	17.75	156.73^b^	111.99^c^
4 mL	57.80^ab^	47.20^ab^	19.80^abc^	16.60^abc^	19.83	22.91	152.55^b^	158.83^b^
Treatment (T)	**		*		NS		***	
Genotype (G)	NS		NS		NS		**	
G*T	NS		NS		NS		**	

**Note:**

Plants were treated every 2 weeks with potassium silicate solutions at concentrations of 0, 2, 3, and 4 mL, mixed with one gallon of water, in five applications during the fall of 2018 and the spring of 2019. Different letters in superscript indicate a statistical difference; the same letter or no letter indicates no significant difference (*P* < 0.05). *, **, *** *P* < 0.05, *P* < 0.01, *P* < 0.001, significance levels, respectively, NS, Non-Significant.

The marketable fruit data (yield/plant) revealed that ‘Flavorfest’ produced higher yields with Si treatments (2 mL (80 g), 3 mL (88 g), and 4 mL (57 g)) than the control (33 g). ‘Rutgers Scarlet™’ marketable fruit yield for 2 mL (82 g) was more than doubled compared to control (32 g), 3 mL (40 g), and 4 mL (47 g) treatments. Overall, the non-marketable yield revealed that 2 mL treatments for ‘Flavorfest’ and ‘Rutgers Scarlet™’ produced more yield than other treatment levels (Table 1).

### Reduced mycelial growth of the *B. cinerea* upon potassium silicate treatments

**Figure 3:**
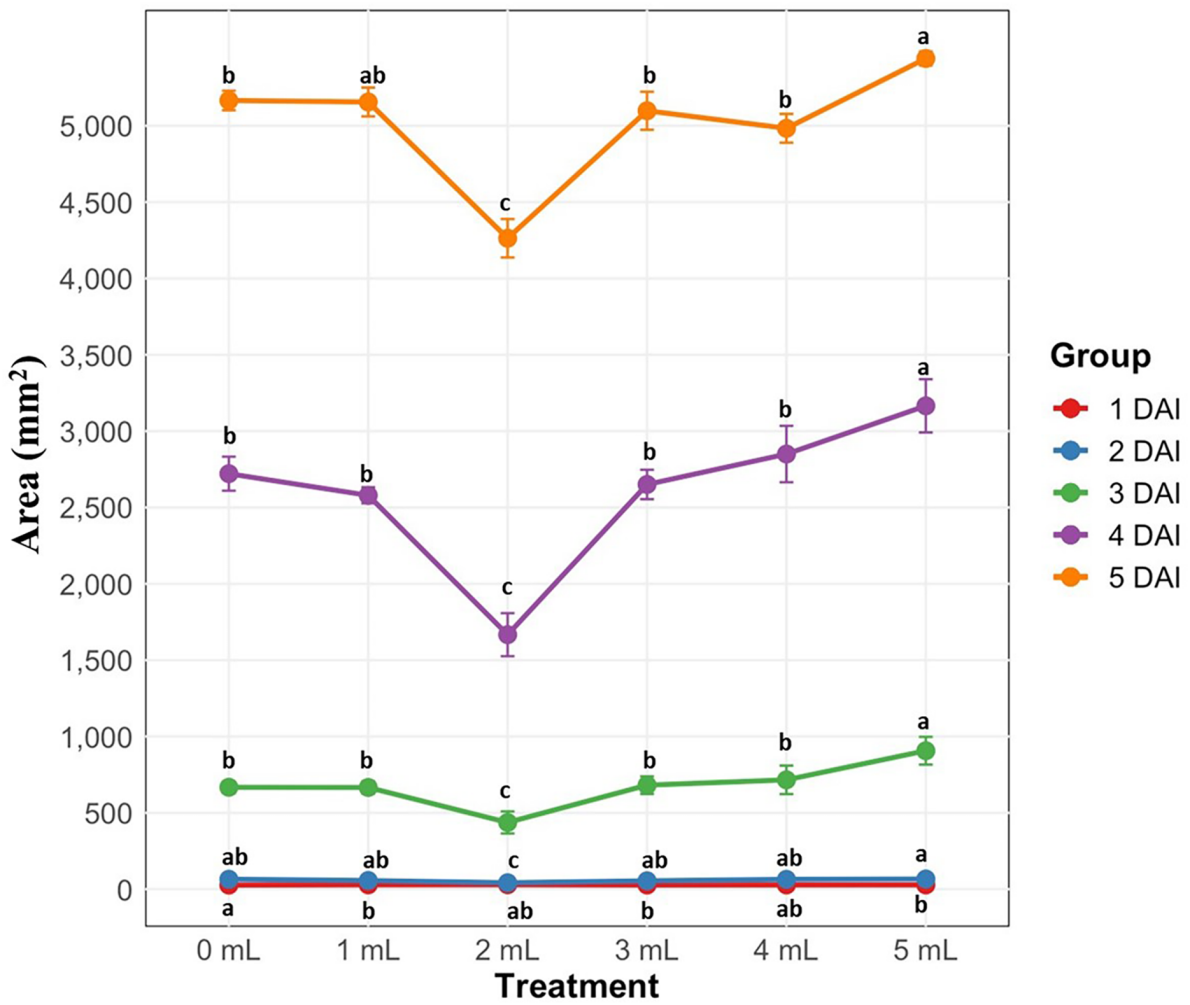
Radial growth of the *B. cinerea* mycelia on petri dishes containing Potato Dextrose Agar (PDA) media supplemented with different concentrations of potassium silicate (0, 1, 2, 3, 4, and 5 mL/gallon). DAI, days after inoculation on fungal plugs. Bars indicate standard errors, with the different letters indicating a statistical difference; the same letter or no letter indicates no significant difference (*P* < 0.05).

The efficacy of K_2_SiO_3_ against *B. cinerea* growth in Potato Dextrose Agar (PDA) was measured daily for 5 days. The statistical interaction among different K_2_SiO_3_ treatments was highly significant (*P* < 0.001). The 2 mL treatment significantly affected the growth area of the fungal mycelium. The substantial differences in radial growth of the fungus were observed from the 2 DAI to 5 DAI upon 2 mL treatment compared to other treatments. From day three, significant differences were observed among the treatments and on day four, 2 mL concentration continued to inhibit mycelium growth (1,667 mm^2^) while the control (2,721 mm^2^), 1 mL (2,580 mm^2^), 3 mL (2,650 mm^2^), 4 mL (2,850 mm^2^), and 5 mL (3,165 mm^2^) mycelium growth increased (Fig. 3). On the last day of observation, 2 mL (4,263 mm^2^) concentration showed a higher inhibition in mycelium growth than all other treatments (5,165–5,440 mm^2^), suggesting the possible effective dose against *B. cinerea* resistance (Fig. 3).

### Postharvest treatments of potassium silicate improve *B. cinerea* fungal resistance in strawberry fruits

**Figure 4:**
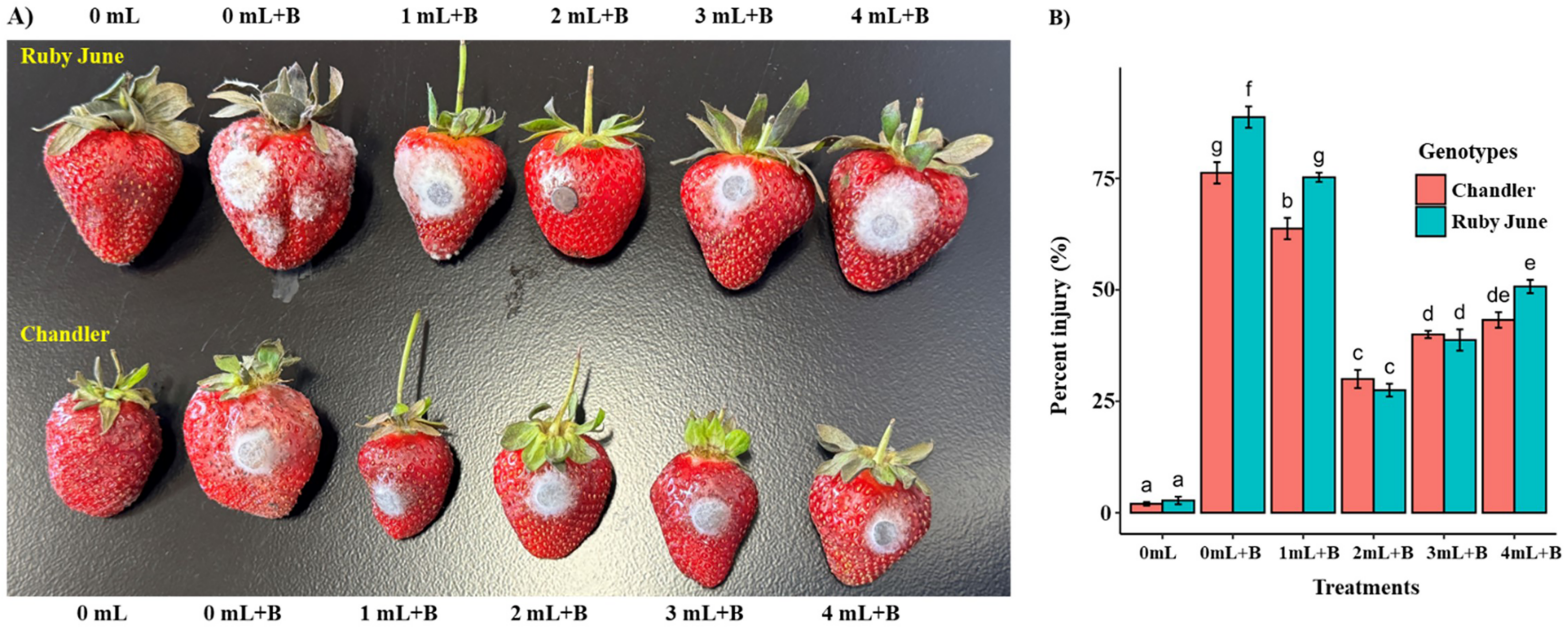
Effect of potassium silicate treatments on postharvest strawberry fruits challenged with *Botrytis cinerea*. The strawberry fruits from two different cultivars, Chandler and Ruby June, were subjected to different concentrations of potassium Si solutions (0, 1, 2, 3, and 4 mL per gallon) and infected with *B. cinerea* fungal discs. Control fruits were maintained without potassium Si treatments and fungal infection. (A) The photographs represent the efficacy of potassium Si treatments in strawberry fruits challenged with *B. cinerea* after 5 days of post-infection. (B) The bar graph shows the percent injury damage caused by *B. cinerea* infection in strawberry fruits treated with different concentrations of potassium Si. 0 mL, control treatment without potassium Si and fungal infection; 0 mL+B, without potassium Si but infected with *B. cinerea*; 1, 2, 3, and 4 mL +B, different concentrations of potassium Si treatments plus *B. cinerea* infection. Bars indicate standard errors, with the different letters indicating a statistical difference; the same letter or no letter indicates no significant difference (*P* < 0.05).

To confirm the efficacy of potassium silicate observed in our *in vitro* studies on *Botrytis cinerea* inhibition, we performed post-harvest treatments of potassium silicate on strawberry fruits to study efficacy on fungal infection and fruit rot. The interaction between strawberry genotypes and treatment was significant in reducing *B. cinerea* fungal growth and fruit rot percent injury in postharvest fruits. The fruits treated with K_2_SiO_3_ had a significantly (*P* < 0.05) lower percent injury compared to *B. cinerea* infection alone in both the strawberry cultivars (Fig. 4). However, 2 mL treatment showed a considerably reduced percent injury in both genotypes compared to other treatments, and a differential response in percent injury between the 1, 3, and 4 mL treatments was observed. Postharvest application of different K_2_SiO_3_ treatments in the present study considerably provided the *B*. *cinerea* resistance in strawberry fruits. The 2 mL treatment has been significant compared to other treatments with less percent injury (Fig. 4).

### Expression of Si transporter genes in postharvest strawberry fruits treated with K_2_SiO_3_ and *B. cinerea* infection

**Figure 5:**
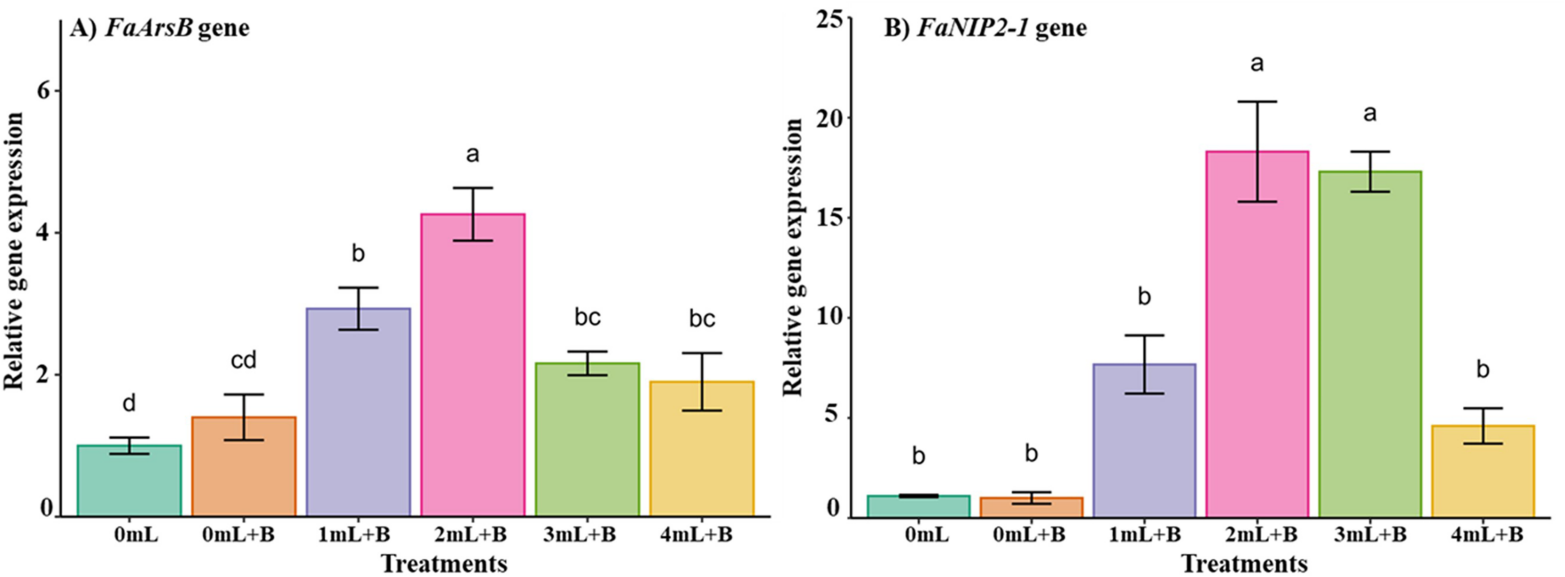
Relative expression of silicon transporter genes in post-harvest strawberry fruits of Chandler cultivar upon potassium Si and *B. cinerea* treatments. (A) The *FaArsB* gene expression and (B) *FaNIP2-1* gene expression in post-harvest fruits after 5 days of treatments. 0 mL, control treatment without potassium Si and fungal infection; 0 mL+B, without potassium Si but infected with *B. cinerea*; 1, 2, 3, and 4 mL +B, different concentrations of potassium Si and *B. cinerea* infection. Bars indicate standard errors, with the different letters indicating a statistical difference; the same letter or no letter indicates no significant difference (*P* < 0.05).

To understand the molecular mechanism involved in the efficacy of potassium Si treatments for improving *B. cinerea* resistance in postharvest strawberry fruits, the expression of silicon transporter genes *FaArsB* and *FaNIP2-1* (Nod26-like major intrinsic protein (aquaporin)) was investigated in the present study in one of the strawberry cultivars, Chandler. The results illustrated that the *FaArsB* gene’s relative expression was significantly higher at 2 mL treatments compared to other K_2_SiO_3_ and non-silicate treatments (Fig. 5). The other silicon transporter gene, *FaNIP2-1*, exhibited significantly higher expression under 2 and 3 mL treatments and maintained moderate expression at 1 and 4 mL treatments compared to both the *B. cinerea* alone and non-silicate treatments (Fig. 5).

## Discussion

Strawberries are high value horticultural crop with significant nutritional and economic importance. However, their productivity as soft plants is often limited by their susceptibility to both abiotic and biotic factors, highlighting the need for approaches to strengthen the plant vigor and disease resistance (Durmuş, Ayyıldız & Erdal, 2025; Han et al., 2026; Limmada et al., 2026; Nazir et al., 2015; Samtani et al., 2012). Among the biotic factors, gray mold, caused by the *Botrytis cinerea*, is devastating, leading to significant yield and postharvest losses. Gray mold disease management largely relies on repeated applications of chemical fungicides, raising concerns about chemical residues, environmental contamination, and the development of pathogen resistance to fungicides. These limitations underscore the need for safer chemical alternatives and sustainable plant disease management (Gulzar et al., 2025a; Hassan et al., 2021; Petrasch et al., 2019).

Silicon has emerged as a promising option in agriculture due to its non-toxic nature and is recognized as an organic compound for plant production because of its role in strengthening plant structural and physiological defenses (Dash et al., 2025; Nimita et al., 2026). Silicon treatments have been reported to improve plant growth and mitigate abiotic and biotic stress by reinforcing cell walls, modulating defense responses, and improving overall plant resistance (Xiao, Li & Jeong, 2022; Xu et al., 2023). Therefore, silicon-based supplementation provides an eco-friendly approach to improving crop resilience and reducing reliance on conventional fungicides (Elshahawy, Saied & Abd-El-Kareem, 2023; Hamrani et al., 2024; Jalal et al., 2024; Saito, Wang & Xiao, 2025). In the present study, the greater accumulation of Si and phytolith formation at 2 mL treatment in the leaf samples may help strengthen the growth, structure, and health of the aerial parts.

SEM-EDS analysis revealed that Si accumulates more in strawberry leaves with 2 mL treatment and is less translocated to the roots than with other Si treatments, providing qualitative and semi-quantitative confirmation of silica accumulation and transport in plant parts. There are no comprehensive studies on the absorption and translocation of Si in strawberry plants and their ratios in shoots and roots (Ouellette et al., 2017). Therefore, the present study is focused on assessing the localization of Si in the leaves and roots of the strawberry plants upon different concentrations of Si foliar spray treatments. Various methods are available for qualitative and quantitative analysis of Si presence in plant samples (Snyder, 2001). However, among these, visualization methods with quantifications provide a simple and reliable approach to assessing the localization of Si in various plant parts (Mundada et al., 2022; Tihlaříková, Neděla & Đorđević, 2019). Similar visualization observations resulting from SEM-EDS image analysis in the present study assist in understanding the mechanisms of absorption and translocation of Si to various plant parts of the strawberry.

Si application-induced phytolith formation in plants is reported to strengthen the plant tissues and act as a mechanical barrier against pests and regulations of nutrient uptake, and enhance abiotic and biotic stress tolerance (Kovács, Kutasy & Csajbók, 2022; Manokari et al., 2023). Higher accumulation of Si upon foliar spray application in plants was reported at the whole plant level, and an enhanced accumulation of Si concentration was observed in leaves (Ouellette et al., 2017; Pilon, Soratto & Moreno, 2013). Quantification of Si using both SEM-EDS and spectrophotometric analysis in our study provided insight into the optimal Si treatment combination and Si partition across different plant tissues, consistent with previous studies (Hajiboland et al., 2018; Mundada et al., 2022; Xu et al., 2023). Upon Si treatment, higher Si accumulation in leaf tissues under the 2 mL treatment strengthened morpho-physiological and yield traits (Xu et al., 2023).

Several researchers have reported the beneficial effects of Si in improving plant growth, strengthening morphological structures, and physiological functions. In our research, the Si treatment significantly enhanced plant width in strawberry plants and phytolith formation. The following studies also reported that Si treatments significantly improved vegetative growth in strawberry plants (Hajiboland et al., 2018; Li et al., 2020; Zahedi et al., 2020) and other plants (Abdou et al., 2022; Sabir et al., 2024). The differential response to the K_2_SiO_3_ treatments in strawberry plants was observed for improved plant physiological traits, such as leaf area, root and shoot growth, and biomass (Dehghanipoodeh et al., 2018; Hajiboland et al., 2018; Laane, 2018; Xiao, Li & Jeong, 2022; Wang & Galletta, 1998).

In the present study, Si treatments significantly enhanced the marketable fruit yield, especially the 2 mL/gallon treatment. Similarly, different Si treatments in strawberries and other fruit crops were reported to increase the fruit yield (Abd-El-Kareem, Elshahawy & Abd-Elgawad, 2019; Laane, 2018; Miyake & Takahashi, 1986). However, most studies conducted were under controlled environmental conditions (Miyake & Takahashi, 1986; Park et al., 2018; Xiao, Li & Jeong, 2022; Xu et al., 2023; Yaghubi et al., 2016) or open fields (Abd-El-Kareem, Elshahawy & Abd-Elgawad, 2019). Our study evaluated the effective concentration of Si foliar spray application for strawberry production and overall plant health under high tunnel conditions. Nevertheless, not much information is available on studies related to foliar spray application under high tunnel conditions (Ouellette et al., 2017). Previous studies in strawberry plants reported the improvement of both quantitative and qualitative traits upon Si treatment (Hajiboland et al., 2018). Similarly, in the present study, the Si application helped strawberry plants to achieve improved morphophysiological characteristics (Fig. S5). Higher accumulation of Si upon 2 mL foliar treatment might help strawberry plants improve their agronomic traits and yield parameters. Still, more field studies are needed to understand the dose-dependent efficacy on various agronomic traits and physiological functions in strawberries.

Earlier *in vitro* studies reported the K_2_SiO_3_ antifungal activity against several phytopathogenic fungi grown on PDA media (Bekker, Kaiser & Labuschagne, 2009; Vidal-Vergara et al., 2022). Similarly, the antifungal activity of K_2_SiO_3_ and significant reduction of fungal mycelial growth were reported in fungal isolates collected from apples. Also, silicon dioxide nanoparticles and other silica forms showed reduced mycelial growth of *B. cinerea* under *in-vitro* conditions (Baka & El-Zahed, 2022; Elshahawy, Saied & Abd-El-Kareem, 2023; Youssef, Roberto & de Oliveira, 2019). Likewise, previous studies reported the antifungal efficacy of Si against *B. cinerea* in several crop plants using fruit assays (Mbili, Laing & Yobo, 2020; Nieto-Angel et al., 2022). In the present study, the reduced mycelial growth of the *B. cinerea* upon 2 mL K_2_SiO_3_treatment corroborates with the prior reports of mycelial growth reduction upon different forms of Si treatments in various fungal species under *in-vitro* conditions (Bekker, Kaiser & Labuschagne, 2009; Elshahawy, Saied & Abd-El-Kareem, 2023; Mbili, Laing & Yobo, 2020). However, further studies are needed to unravel the mechanism of the antifungal functions of potassium Si against *B. cinerea*.

Postharvest applications of potassium silicate have been reported in several horticultural fruit crops, including small fruits such as strawberries and blueberries, to maintain the fruit quality and reduce the different fungal diseases, including gray mold (Elshahawy, Saied & Abd-El-Kareem, 2023; Hamrani et al., 2024; Jalal et al., 2024; Mahendranathan & Adikaram, 2019; Moscoso-Ramírez & Palou, 2014; Nikagolla, Udugala-Ganehenege & Daundasekera, 2019; Saito, Wang & Xiao, 2025; Tesfay, Bertling & Bower, 2011). However, there were no comprehensive reports on strawberries upon postharvest application of K_2_SiO_3_ against *B. cinerea* infection using the immersion method (Lopes et al., 2014). With this, the current study opens an avenue to explore protection strategies against gray mold fungus with postharvest dipping applications of K_2_SiO_3_.

In several plant species, the genes *ArsB* and *NIP2-1* were reported as membrane transporters that play an important role in silicon uptake and other functions (Hu, Cai & Jeong, 2019; Jadhao, Bansal & Rout, 2020; Ouellette et al., 2017). In the present study, K_2_SiO_3_ treatments significantly enhanced the expression of silicon transporter genes. Specifically, both the genes (*ArsB* and *NIP2-1*) showed higher expression at 2 mL treatment, and differential response of the gene expressions were observed under other K_2_SiO_3_ treatments (Fig. 5). The differential expression of silicon transporter genes upon K_2_SiO_3_ treatments was observed in different plant species suggesting the response various with the treatments and plant species (Chaiwong et al., 2022). In this study, 2 mL treatment enhanced gene expression and improved *B. cinerea* resistance in postharvest strawberry fruits. This suggests the possible mechanism of induced expression of silicon transporter genes conferring resistance to fungal incidence upon K_2_SiO_3_ treatment. Similar reports have been documented in the plant species, the positive correlation between the K_2_SiO_3_ treatments, induced gene expression of silicon transporter genes, and fungal disease incidences (Chaiwong et al., 2022; Jadhao, Bansal & Rout, 2020). Overexpression of Si transporter genes and induced expression upon Si treatment in plants led to significantly higher Si accumulation; consequently, plants acquired resistance to fungal and different pathogens (Fauteux et al., 2005; Jadhao, Bansal & Rout, 2020; Li et al., 2022; Vivancos et al., 2015), a similar mechanism possibly helping achieve *Botrytis* resistance in strawberries, as evidenced by the enhanced expression of si transporter genes upon treatment. Our work reports the promising nature of K_2_SiO_3_ treatments *via* foliar application, *in vitro* antifungal activity, and post-harvest applications as sustainable strategies to enhance crop growth and disease management.

## Conclusion

Our SDS-EMS analysis revealed the variations in the distribution pattern and accumulation levels of silica across the leaves and roots among the treatments. Quantitative analysis of Si concentrations further confirmed a higher accumulation of Si in strawberry leaf samples at 2 mL treatment compared to other doses. The study, conducted under high tunnel conditions, revealed that different concentrations of K_2_SiO_3_ foliar applications had variable effects on Si accumulation in different cultivars. The 2 mL per gallon treatment showed consistent impact on both yield and plant width improvement in strawberry cultivars. However, higher treatment concentrations (3 and 4 mL) showed reduced or nonsignificant effects on the traits studied.

**Figure 6:**
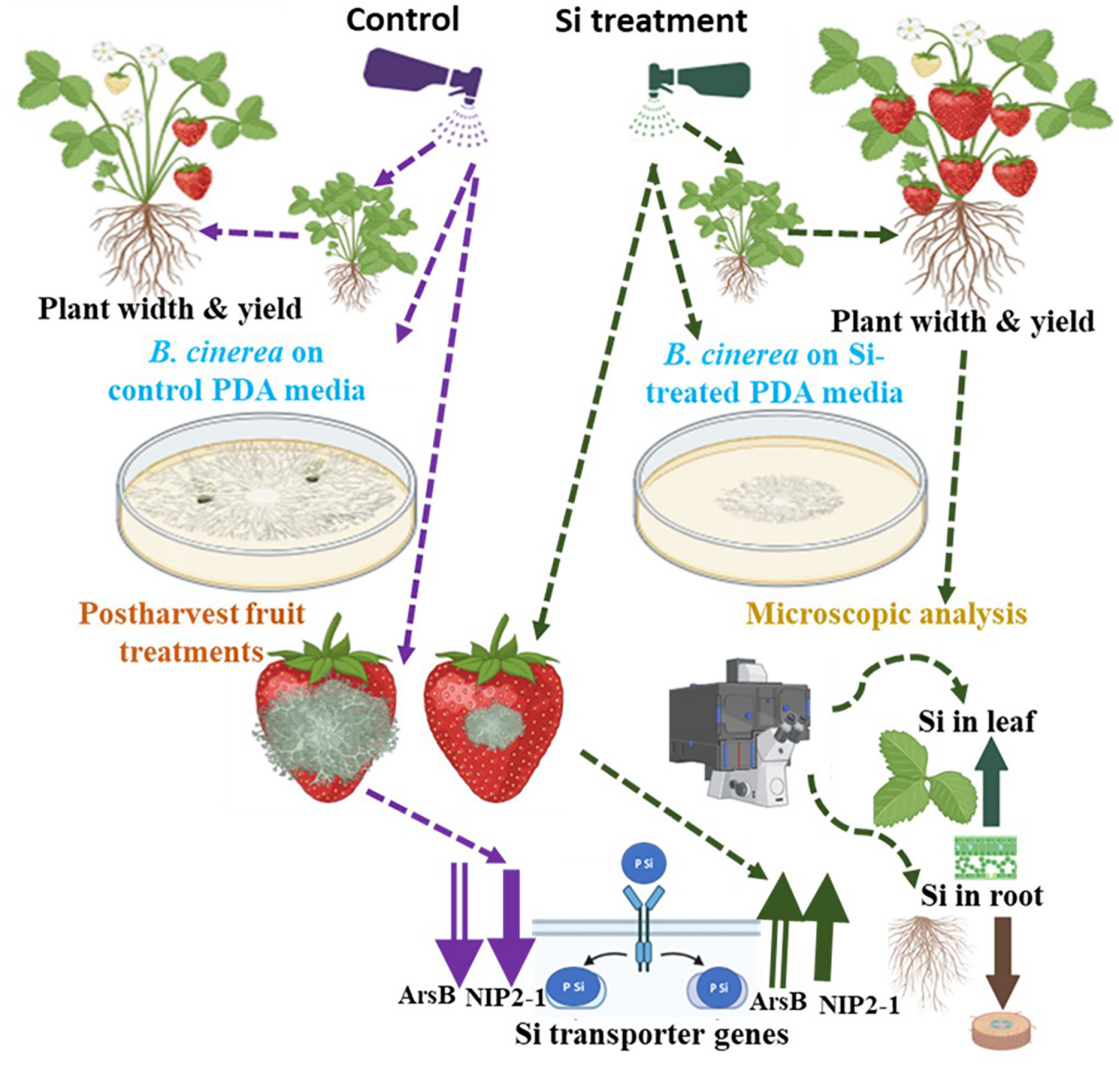
Overview of the potassium silicate efficacy in improving the strawberry agronomic traits and combating *Botrytis cinerea* infection. The effect of potassium silicate treatments compared to control (water or without treatment) on strawberry plants’ width and yield, Si accumulation in leaves and roots, and the antifungal activity and post-harvest fruit protection from *B. cinerea* infection and Si transporter gene expression. The purple (control) and green dotted arrows (Si treatments) represent the effects of the control and Si treatments, respectively, on various traits. The direction of the thick arrows indicates si concentrations and gene expression patterns. The figure was drawn using Biorender software (https://www.biorender.com/).

Our *in-vitro* studies demonstrated the preventive antifungal properties of K_2_SiO_3_, reducing the radial growth area of *B. cinerea* fungal mycelia. The 2 mL treatment was shown to be efficient in reducing the *B. cinerea* infection on postharvest strawberry cultivars compared to other treatments and enhanced expression of silicon transporter genes, showing a possible gene responsible for si transport and promoting the si accumulation and treatment efficiency (Fig. 6). These findings emphasize the potential effect of K_2_SiO_3_ in strengthening agronomic traits in strawberry crop production and postharvest management. The use of K_2_SiO_3_ in strawberry production offers an alternative to fungicides and synthetic fertilizers, making it a cost-effective and sustainable approach. Further, more comprehensive, multi-year field studies are needed to understand the potential benefits of treatments for crop improvement and their impact on the environment. Also, functional validation of molecular pathways to understand the impact of K_2_SiO_3_ on various agronomic traits and *B. cinerea* resistance.

## Supplemental Information

10.7717/peerj.21151/supp-1 Supplemental Information 1 The soil test report before the potassium silicate treatments from the field soil under high tunnels.

10.7717/peerj.21151/supp-2 Supplemental Information 2 The soil test report after the potassium silicate treatments and harvest.

10.7717/peerj.21151/supp-3 Supplemental Information 3 SEM-EDS images showing the Si distribution in strawberry root samples upon different concentrations of Si treatments. The Scanning Electron Microscopy images coupled with Energy-Dispersive X-ray Spectrometry (SEM-EDS) analysis were used to see the distribution of different elements in the root tissues. (A) Black and white images, shows that the white particles are silica in root samples. (B) The elemental maps of orange particles as silica and blue particles as potassium (K). (C) The elemental maps represent the orange particles of silica alone. (D) The elemental maps represent the overlay of all particles, silica (Si, orange), potassium (K, blue), gold (Au, gold), and oxygen (O, pink), as indicated. Maps collected at 10kV over the SEM image. Image width=100 µm. E) EDS spectra represent the Si and other ‘elements’ peak area in strawberry root samples upon different concentrations of potassium silica treatments: control (0 mL), 2 mL treatment, 3 mL treatment, and 4 mL treatment.

10.7717/peerj.21151/supp-4 Supplemental Information 4 Silica content in strawberry plant samples treated with different concentrations of potassium-silicate. The bar graph shows the Si concentrations in leaf samples (A) and root samples (B) measured using spectrophotometer analysis. The Si concentrations were analyzed from leaf and root samples collected in the fall of 2018.

10.7717/peerj.21151/supp-5 Supplemental Information 5 Effect of potassium silicate treatments on morphophysiological traits in strawberry plants. (A) Plant canopy variation upon different concentrations of Si treatments (0, 2, 3, and 4 mL/gallon) on strawberry plants. (B) Fruit size upon different concentrations of Si treatments (0, 2, 3, and 4 mL/gallon).

10.7717/peerj.21151/supp-6 Supplemental Information 6 List of the strawberry gene primers used in the Quantitative real-time PCR (RT-qPCR).

10.7717/peerj.21151/supp-7 Supplemental Information 7 MIQE Checklist.

10.7717/peerj.21151/supp-8 Supplemental Information 8 Raw data.

## Additional Information and Declarations

### Data Availability

The following information was supplied regarding data availability:

The raw data is available in the Supplemental File.
