# Supplementary figures and images for: Investigating potassium silicate efficacy and mechanisms for improving the strawberry agronomic traits and gray mold fungal resistance
Source: PeerJ. 2026 Apr 29;14:e21151. doi: 10.7717/peerj.21151 (PMC13135329; doi:10.7717/peerj.21151)

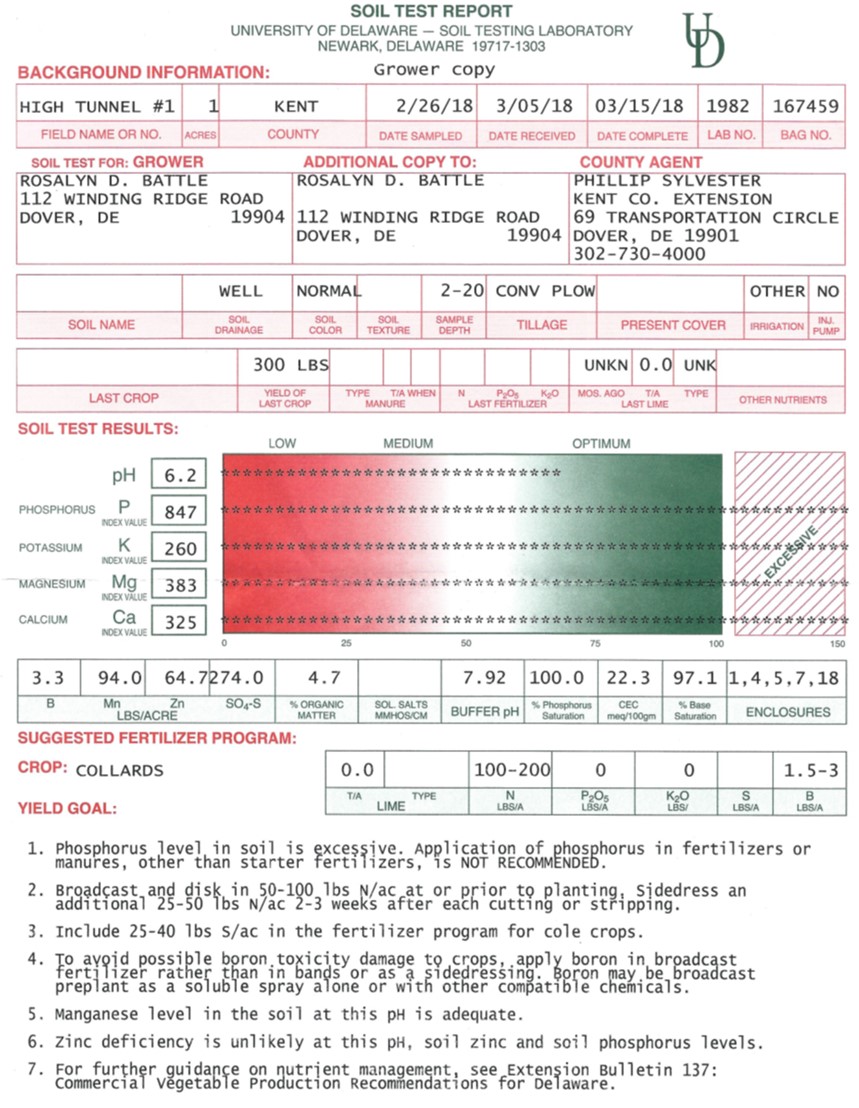

Supplement: Supplemental Information 1 [file peerj-14-21151-s001.jpg]

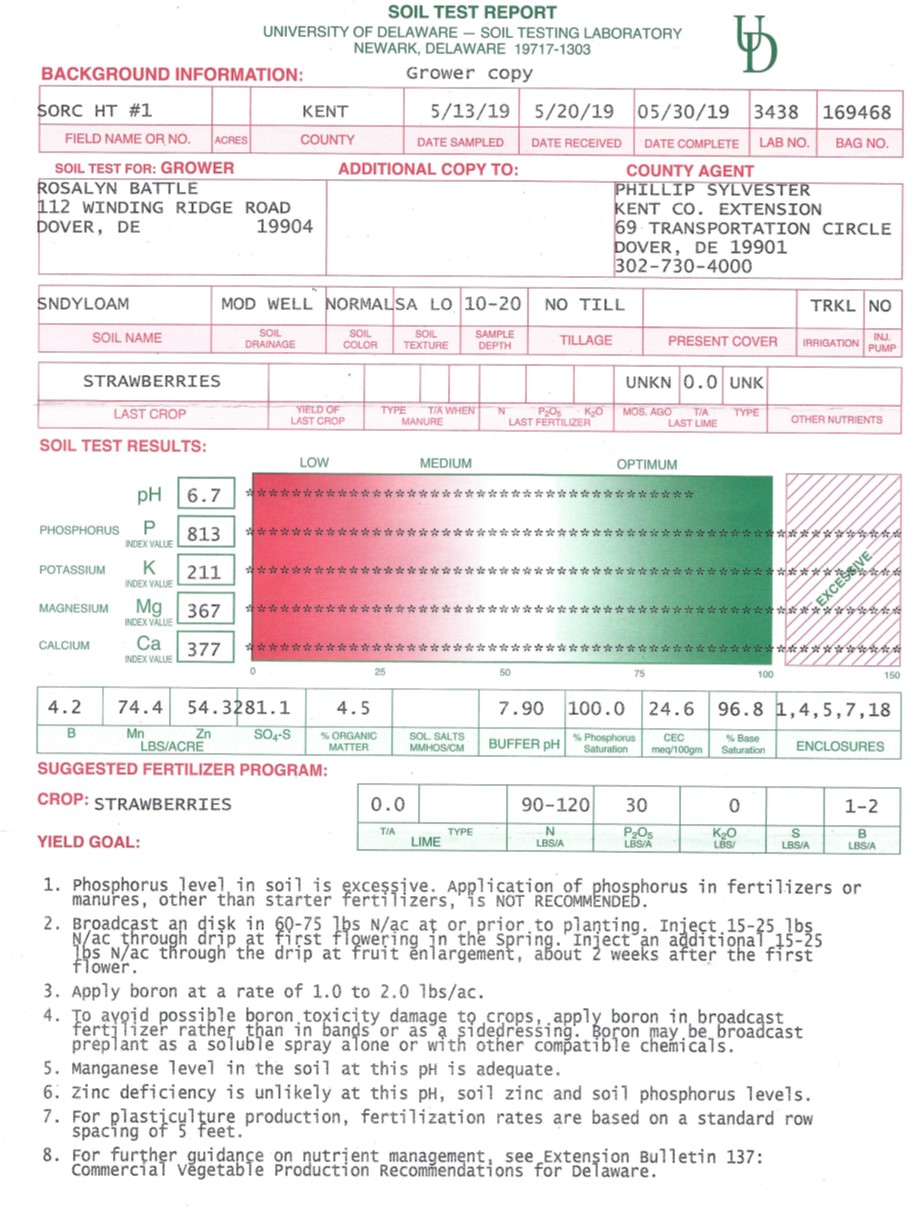

Supplement: Supplemental Information 2 [file peerj-14-21151-s002.jpg]

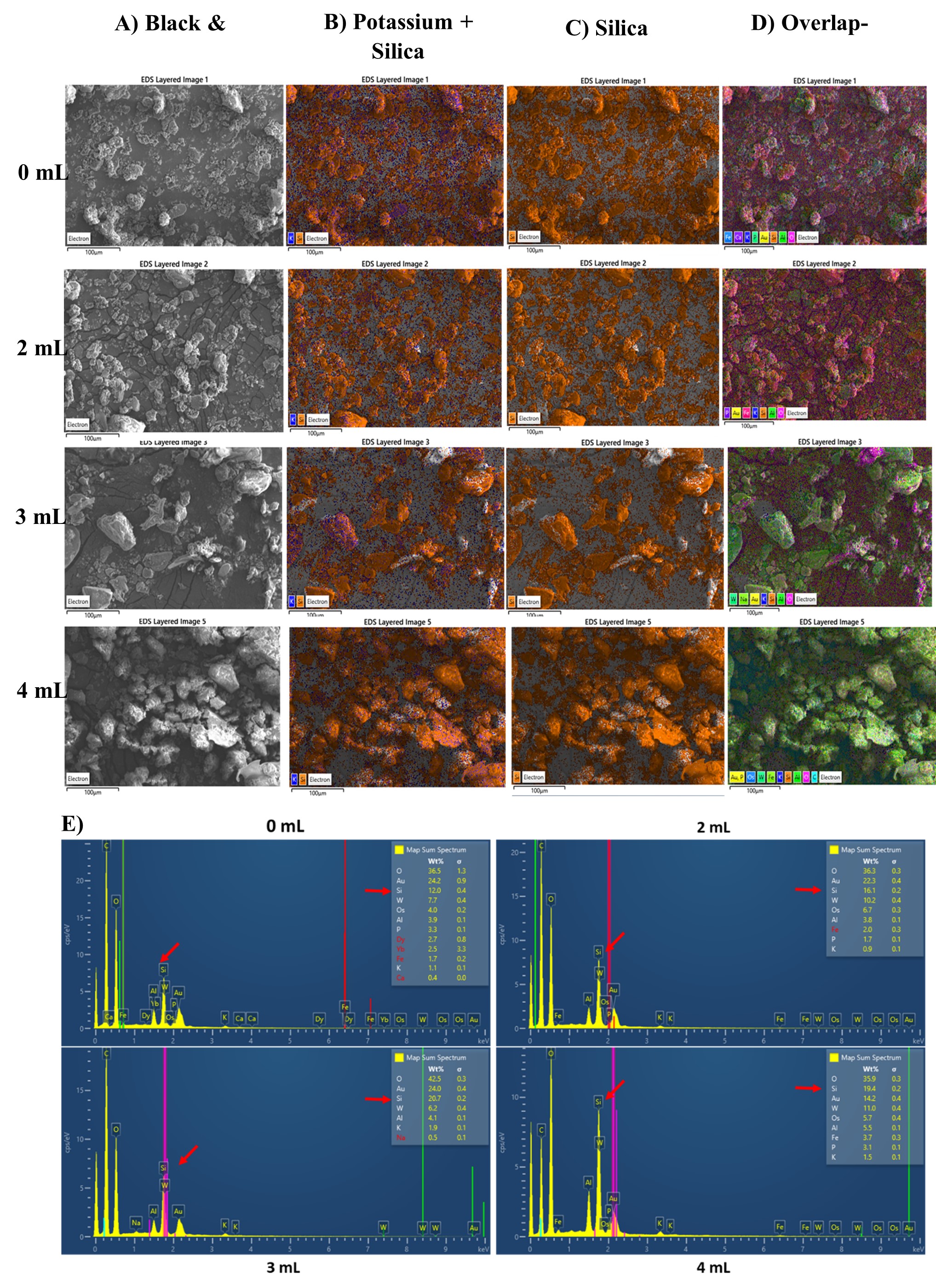

Supplement: Supplemental Information 3 — The Scanning Electron Microscopy images coupled with Energy-Dispersive X-ray Spectrometry (SEM-EDS) analysis were used to see the distribution of different elements in the root tissues. (A) Black and white images, shows that the white particles are silica in root samples. (B) The elemental maps of orange particles as silica and blue particles as potassium (K). (C) The elemental maps represent the orange particles of silica alone. (D) The elemental maps represent the overlay of all particles, silica (Si, orange), potassium (K, blue), gold (Au, gold), and oxygen (O, pink), as indicated. Maps collected at 10kV over the SEM image. Image width=100 µm. E) EDS spectra represent the Si and other ‘elements’ peak area in strawberry root samples upon different concentrations of potassium silica treatments: control (0 mL), 2 mL treatment, 3 mL treatment, and 4 mL treatment. [file peerj-14-21151-s003.jpg]

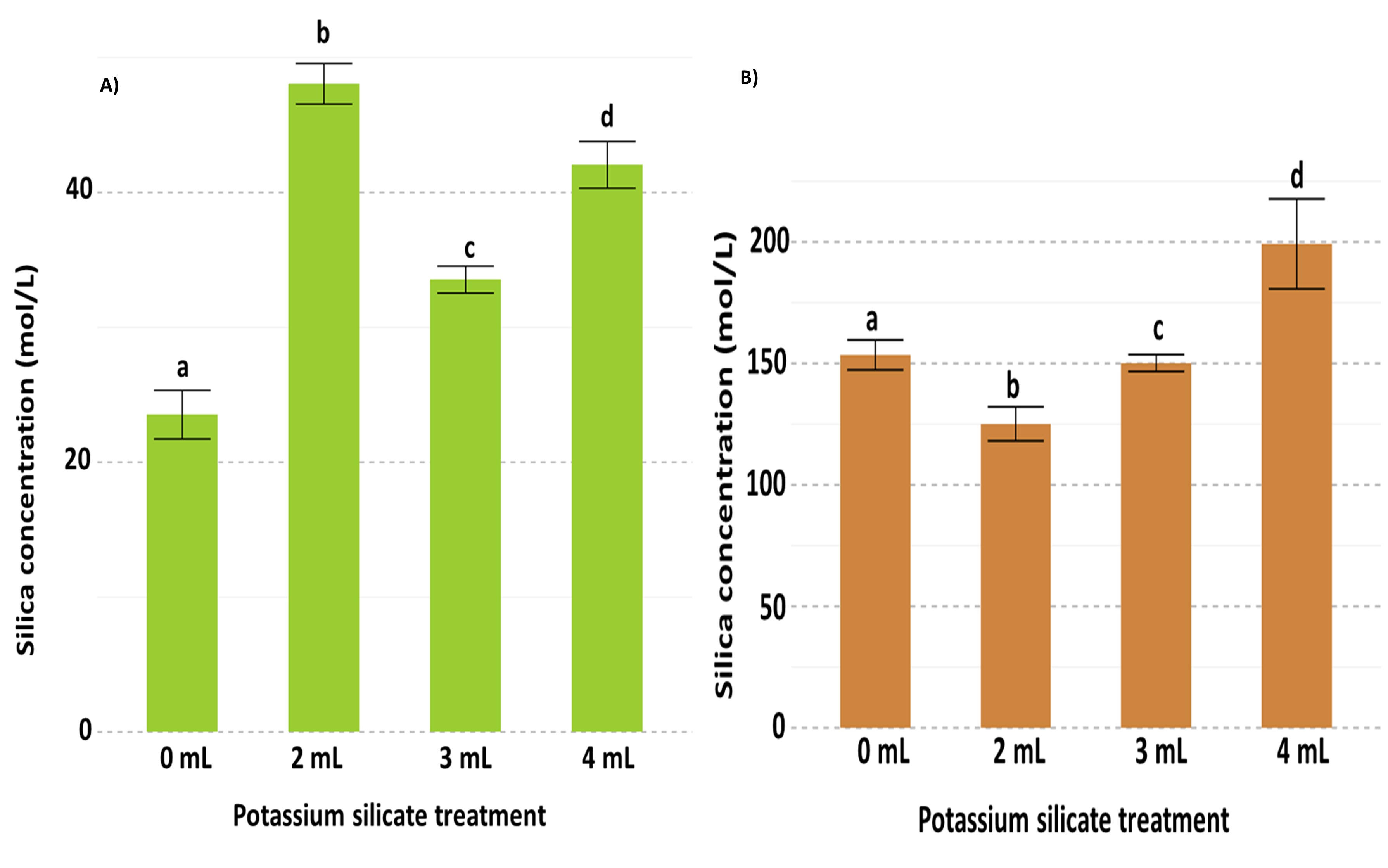

Supplement: Supplemental Information 4 — The bar graph shows the Si concentrations in leaf samples (A) and root samples (B) measured using spectrophotometer analysis. The Si concentrations were analyzed from leaf and root samples collected in the fall of 2018. [file peerj-14-21151-s004.jpg]

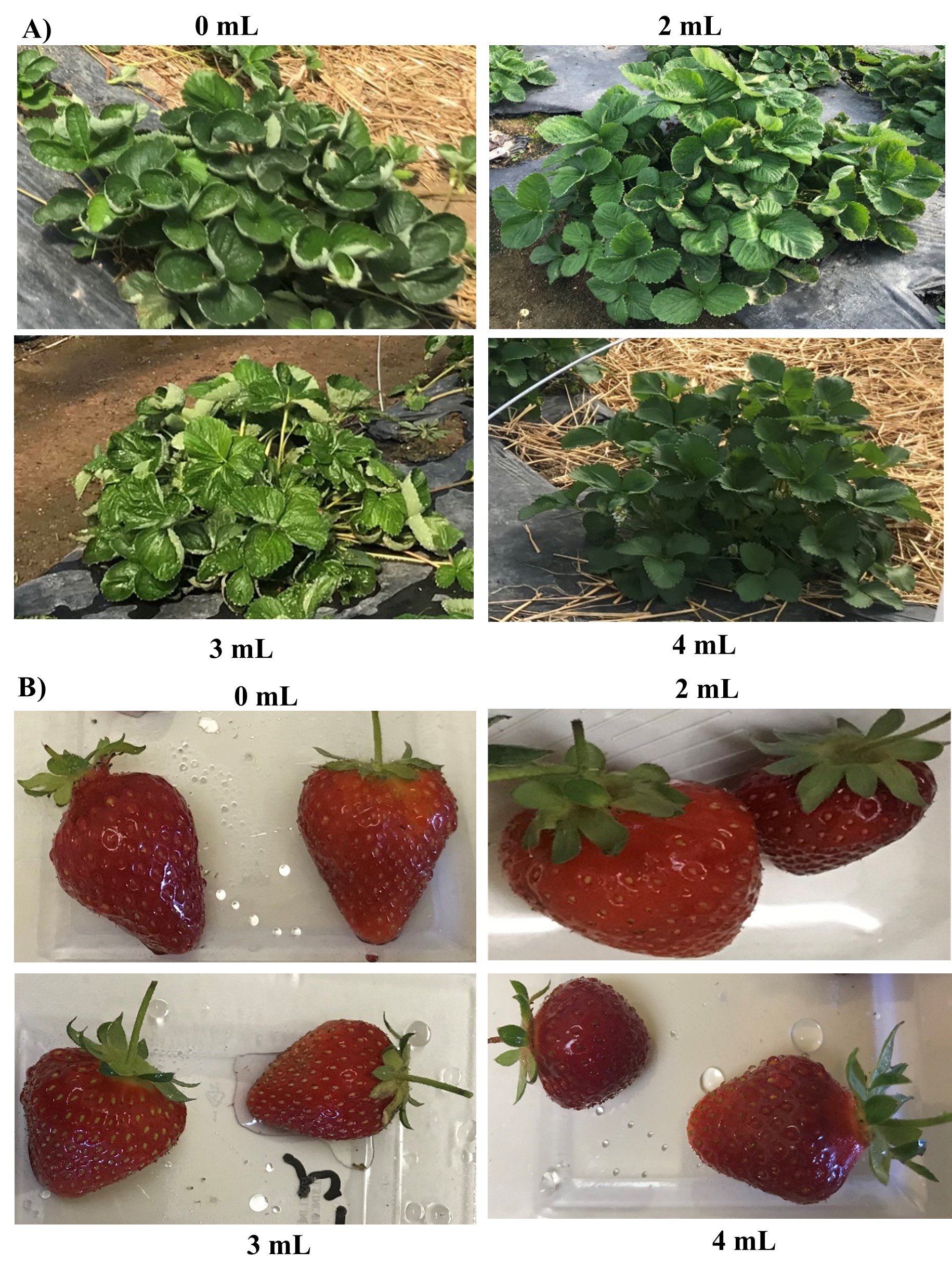

Supplement: Supplemental Information 5 — (A) Plant canopy variation upon different concentrations of Si treatments (0, 2, 3, and 4 mL/gallon) on strawberry plants. (B) Fruit size upon different concentrations of Si treatments (0, 2, 3, and 4 mL/gallon). [file peerj-14-21151-s005.jpg]
