# Supplementary material for: Investigating potassium silicate efficacy and mechanisms for improving the strawberry agronomic traits and gray mold fungal resistance
Source: PeerJ. 2026 Apr 29;14:e21151. doi: 10.7717/peerj.21151 (PMC13135329; doi:10.7717/peerj.21151)
Supplement: Supplemental Information 6 [file peerj-14-21151-s006.docx]

| **Gene name** | **Forward sequence (5’ – 3’)** | **Reverse sequence (5’ – 3’)** |
| --- | --- | --- |
| *FaGAPDH2* | CCCAAGTAAGGATGCCCCCATGTTCG | TTGGCAAGGGGAGCAAGACAGTTGGTAG |
| *FaArsB* | GCGGAGGAGGATGTGAGTTC | TCAGTTGAAGGCACACGGTT |
| *FaNIP2-1* | CCGTCGCCACAGATACTAAA | TCATGGAGCCACCTGATATTG |

**Supplementary Table 1** List of the strawberry gene primers used in the Quantitative real-time PCR (RT-qPCR).
