# Supplementary material for: Investigating potassium silicate efficacy and mechanisms for improving the strawberry agronomic traits and gray mold fungal resistance
Source: PeerJ. 2026 Apr 29;14:e21151. doi: 10.7717/peerj.21151 (PMC13135329; doi:10.7717/peerj.21151)
Supplement: Supplemental Information 7 [file peerj-14-21151-s007.docx]

| ITEM TO CHECK | IMPORTANCE | DETAILS |
| --- | --- | --- |
| **EXPERIMENTAL DESIGN** |  |  |
| Definition of experimental and control groups | E | The experimental group consisted of control, fungus (infected with B. cinerea), and different concentrations of K2SiO3 (0, 1, 2, 3, and 4 mL per gallon) with fungal infection groups. Control fruits were maintained without potassium Si treatments and fungal infection. RT-qPCR was performed to assess the function of the silicon transporter genes in one of the strawberry cultivars, Chandler (Line 185-208). |
| Number within each group | E | Three samples for each group were used (Lines 205-217). |
| Assay carried out by core lab or investigator's lab? | D | The assays were carried out in the investigator's lab, and quantitative gene expression analysis was performed at the Ag Annex Building, Department of Agriculture and Natural Resources, Delaware State University (Line 215-216). |
| Acknowledgement of authors' contributions | D | Authors' contributions are provided in the manuscript, and their contributions are acknowledged in the gene expression work ( Lines 426- 431). |
| **SAMPLE** |  |  |
| Description | E | Flash frozen fruit samples were used for RNA extraction from one of the strawberry cultivars, Chandler (Lines 184-221） |
| Volume/mass of sample processed | D | 100 mg for fresh frozen tissue used for RNA isolation（Lines 207-210). |
| Microdissection or macrodissection | E | Not applicable (NA). |
| Processing procedure | E | The sample processing procedure and RNA extraction protocol cited in the manuscript (Lines 209-210). |
| If frozen - how and how quickly? | E | Flash-frozen fruit samples from one of the strawberry cultivars, Chandler, were used for RNA extraction (Lines 203-204）. |
| If fixed - with what, how quickly? | E | NA |
| Sample storage conditions and duration (especially for FFPE) | E | Flash frozen fruit samples were stored at -80°C until used for extraction. (Lines 204-205 and 209-210). |
| **NUCLEIC ACID EXTRACTION** |  |  |
| Procedure and/or instrumentation | E | RNA extraction was performed using the Spectrum Plant Total RNA Kit and estimated using the Nanodrop 2000/2000C (Thermo Scientific) located in the J.W. Baker Building, Department of Agriculture and Natural Resources, Delaware State University (Line 207-212). |
| Name of kit and details of any modifications | E | RNA extraction was performed using the Spectrum Plant Total RNA Kit (Sigma-Aldrich, St Louis, MO, USA) (Line 207-210）. |
| Source of additional reagents used | D | Additional reagents were sourced from Thermofisher and Sigma Aldrich. |
| Details of DNase or RNAse treatment | E | The genomic DNA was removed using the DNase treatment according to the protocol (Line 207-210). |
| Contamination assessment (DNA or RNA) | D | Contamination was assessed using agarose gel electrophoresis and semiquantitative RT-PCR with gene-specific and housekeeping gene primers, utilizing a cDNA template. No RT (RNA template) was used as a negative control. |
| Nucleic acid quantification | E | Quantification was assessed using a NanoDrop spectrophotometer. |
| Instrument and method | E | RNA quantity and quality were assessed using a NanoDrop spectrophotometer. |
| Purity (A260/A280) | D | RNA purity was assessed by measuring the A260/A280 ratio, aiming for values between 1.8 and 2.0. |
| Yield | D | RNA yield was assessed using a NanoDrop spectrophotometer. |
| RNA integrity method/instrument | E |  |
| RIN/RQI or Cq of 3' and 5' transcripts | E |  |
| Electrophoresis traces | D | RNA quality was assessed using an agarose gel iBright imaging system, Thermo Fisher. |
| Inhibition testing (Cq dilutions, spike or other) | E | Specific inhibition testing (e.g., Cq dilutions, spike) was not performed. However, the Power SYBR Green Master Mix was used according to the manufacturer’s guidelines, and all reactions were performed in triplicate to ensure the accuracy and reproducibility of the results. No significant inhibition was observed in the amplification process. |
| **REVERSE TRANSCRIPTION** |  |  |
| Complete reaction conditions | E | Reverse transcription reaction performed using Applied Biosystems™ Power SYBR™ Green RNA-to-CT™ 1-Step Kit (Line 217-219). |
| Amount of RNA and reaction volume | E | 0.05 µg of RNA was used in a 15 µL reaction volume. (Line 218-219) |
| Priming oligonucleotide (if using GSP) and concentration | E | Master mix contains all the required concentrations (Line 217-219) |
| Reverse transcriptase and concentration | E | Master mix contains Invitrogen™ ArrayScript™ UP Reverse Transcriptase and all other required components in Power SYBR™ Green RNA-to-CT™ 1-Step Kit ((Line 217-219) |
| Temperature and time | E | The reverse transcription reaction was carried out according to the protocol (Line 217-219). |
| Manufacturer of reagents and catalogue numbers | D | Reagents were obtained from Thermo Fisher, Applied Biosystems™ Power SYBR™ Green RNA-to-CT™ 1-Step Kit, Catalog number 4389986. |
| Cqs with and without RT | D |  |
| Storage conditions of cDNA | D |  |
| **qPCR TARGET INFORMATION** |  |  |
| If multiplex, efficiency and LOD of each assay | E | In this study, multiplex assays were not performed. |
| Sequence accession number | E | Sequence accession numbers for target genes are provided in the supplementary materials. ( Supplementary Table 3). |
| Location of amplicon | D | Amplicons were located within across different exons to avoid amplification of genomic DNA. |
| Amplicon length | E | Amplicon lengths ranged from 86 to 156 bp, and the amplicon sizes of the primers are provided in the supplementary materials (Supplementary Table 3). |
| In silico specificity screen (BLAST, etc) | E | Primers were designed using Primer 3 software and  from IDT Primer Quest (Integrated DNA Technologies, Coralville, IA, USA) to ensure specificity. |
| Pseudogenes, retropseudogenes or other homologs? | D |  |
| Sequence alignment | D | Primer sequences were aligned with the reference genome to confirm their specificity. |
| Secondary structure analysis of amplicon | D | Secondary structures were assessed using mFold. |
| Location of each primer by exon or intron (if applicable) | E | Genomic DNA contamination was prevented by using the gDNA Eliminator column during RNA isolation. Primers were designed to span exon-exon junctions, with the intronic regions between exons selected to be large enough to avoid amplification of genomic DNA. |
| What splice variants are targeted? | E | In this study, the largest transcript or the most representative transcript variant for each gene was targeted. |
| **qPCR OLIGONUCLEOTIDES** |  |  |
| Primer sequences | E | Primer sequences were provided in the Supplementary Table 1. |
| RTPrimerDB Identification Number | D | Not applicable. |
| Probe sequences | D | Probe sequences were not required as SYBR Green chemistry was utilized. |
| Location and identity of any modifications | E | No modifications were used. |
| Manufacturer of oligonucleotides | D | Primers were synthesized by Integrated DNA Technologies, Coralville, IA, USA (Line 213). |
| Purification method | D | Primers were purified using Standard Desalting. |
| **qPCR PROTOCOL** |  |  |
| Complete reaction conditions | E | The reaction mixture contained 7.5 2 µL of Power SYBR. Green RNA-to-CT. 1-Step Kit master mix (Applied Biosystems), 0.15 µL of RT enzyme mix, 1.5 µL of each of the forward and reverse primers (5  µL and 50 ng template RNA (2 µL) in a final volume of 15 µL (Line 217-220). |
| Reaction volume and amount of cDNA/DNA | E | NA |
| Primer, (probe), Mg++ and dNTP concentrations | E | Power SYBR™ Green (Applied Biosystems) was used for qRT-PCR, and the reaction was performed following the manufacturer's protocol, as the mix contains optimized concentrations of Mg++, dNTPs, and enzymes. |
| Polymerase identity and concentration | E | Applied Biosystems™ Power SYBR™ Green RNA-to-CT™ 1-Step Kit was used for qRT-PCR, and the reaction was performed following the manufacturer's protocol. The AmpliTaq Gold™ DNA Polymerase UP (Ultra Pure) for hot-start PCR , dNTP mix (with dUTP instead of dTTP), SYBR Green I dye, and MgCl₂, all at optimized concentrations. |
| Buffer/kit identity and manufacturer | E | Applied Biosystems™ Power SYBR™ Green RNA-to-CT™ 1-Step Kit was used (Line 218). |
| Exact chemical constitution of the buffer | D | It includes Applied Biosystems™ AmpliTaq Gold™ DNA Polymerase UP (Ultra Pure), Invitrogen™ ArrayScript™ UP Reverse Transcriptase, a dNTP blend including dUTP, an RNase inhibitor, an additive that reduces primer-dimer formation, and a passive internal reference based on proprietary ROX™ dye, for increased data precision. |
| Additives (SYBR Green I, DMSO, etc) | E | Power SYBR™ Green was included in the Master Mix: Applied Biosystems™ Power SYBR™ Green RNA-to-CT™ 1-Step Kit, Catalog number 4389986. |
| Manufacturer of plates/tubes and catalog number | D | MicroAmp Endura Plate Optical 96-Well Plates and sealers were obtained from Applied Biosystems. |
| Complete thermocycling parameters | E | The thermocycler program was set at 95°C for 5 min, followed by 35 cycles of 95°C for 30 s and 60°C for 30 s (Line 220-221). |
| Reaction setup (manual/robotic) | D | Reaction setup was performed manually using pipettes. |
| Manufacturer of qPCR instrument | E | qPCR was conducted using the 7500 Real-Time PCR system (Applied Biosystems, Waltham, MA, USA) according to the manufacturer’s protocol (Lines 214-215). |
| **qPCR VALIDATION** |  |  |
| Evidence of optimisation (from gradients) | D | Gradient PCR was performed to standardize the optimal annealing temperature for primers, ensuring efficient amplification and minimal non-specific binding. |
| Specificity (gel, sequence, melt, or digest) | E | The specificity of the primers was confirmed by melting curve analysis using Applied Biosystem 7500 software and, when necessary, validated by gel electrophoresis of PCR products to confirm the expected amplicon size. |
| For SYBR Green I, Cq of the NTC | E | For SYBR Green, the Cp value of the non-template control was greater than 35 or 40, confirming the absence of contamination. |
| Standard curves with slope and y-intercept | E | Standard curve analysis was not performed, as the analysis was done using the ΔΔCt method, which does not require constructing a standard curve. |
| PCR efficiency calculated from slope | E | As the analysis utilized the ΔΔCt method, which does not require standard curve construction. Hence, it did not perform. |
| Confidence interval for PCR efficiency or standard error | D | PCR efficiency was not calculated using a standard curve, so the confidence interval or standard error of the PCR efficiency was not determined. Still, the software provided it based on the ΔΔCt method. |
| r2 of standard curve | E | As a standard curve was not used for this analysis, the r² value is not applicable. |
| Linear dynamic range | E | Since the ΔΔCt method was applied in this study, the linear dynamic range was not assessed using a standard curve. |
| Cq variation at lower limit | E | No issues were observed with Cq values at low expression. |
| Confidence intervals throughout range | D | As the analysis was performed using the ΔΔCt method, confidence intervals for the entire range were not calculated. However, the assays were optimized for high specificity and reproducibility, ensuring reliable results within the measured gene expression levels. |
| Evidence for limit of detection | E | No issues related to sensitivity or limit in detection were observed, and all measured genes showed detectable expression levels within the dynamic range of the assay. |
| If multiplex, efficiency and LOD of each assay | E | Multiplex assays were not performed in this study. |
| **DATA ANALYSIS** |  |  |
| qPCR analysis program (source, version) | E | qPCR analysis was performed using Applied Biosystem 7500 software using the ΔΔCt method (Line 221-223). |
| Cq method determination | E | Cp values were determined using Applied Biosystem 7500 software (Line 221-223). |
| Outlier identification and disposition | E | An outlier is removed rarely. Triplicate measurements were taken for each sample (Line 217). |
| Results of NTCs | E | NTC (No Template Control) results were confirmed using semiquantitative RT-PCR; no amplification of the genes was observed when RNA was used as the template (no RT). Strict precautions were taken to prevent contamination, including careful handling of primers and reagents, as well as performing the experiments. |
| Justification of number and choice of reference genes | E | The reference gene (FaGADPH2) was chosen because its expression was consistent across all samples, ensuring reliable normalization in the analysis (Line 221-223). |
| Description of normalization method | E | Normalization used a reference gene and control sample expression to account for RNA quantity and quality variations. (Line 221-223). |
| Number and concordance of biological replicates | D | For each treatment, three biological replicates were maintained（Line 217）. |
| Number and stage (RT or qPCR) of technical replicates | E | For each treatment, three biological and three technical replicates were maintained（Line 217）. |
| Repeatability (intra-assay variation) | E |  |
| Reproducibility (inter-assay variation, %CV) | D |  |
| Power analysis | D |  |
| Statistical methods for result significance | E | Statistical significance was determined using One-way ANOVA analysis to compare the treatments. A p-value of less than 0.05 was considered statistically significant. (Lines 226-232) |
| Software (source, version) | E | Data analysis was conducted using R package agricolae (Lines 226-232) |
| Cq or raw data submission using RDML | D | The raw data were submitted in the supplementary file as Excel files. |
