# Supplementary material for: Investigating potassium silicate efficacy and mechanisms for improving the strawberry agronomic traits and gray mold fungal resistance
Source: PeerJ. 2026 Apr 29;14:e21151. doi: 10.7717/peerj.21151 (PMC13135329; doi:10.7717/peerj.21151)
Supplement: Supplemental Information 8 [file peerj-14-21151-s008.zip › Raw data/Fig 5_qPCR-RNA-PCR gels/Gel Images .pptx]

## Slide 1
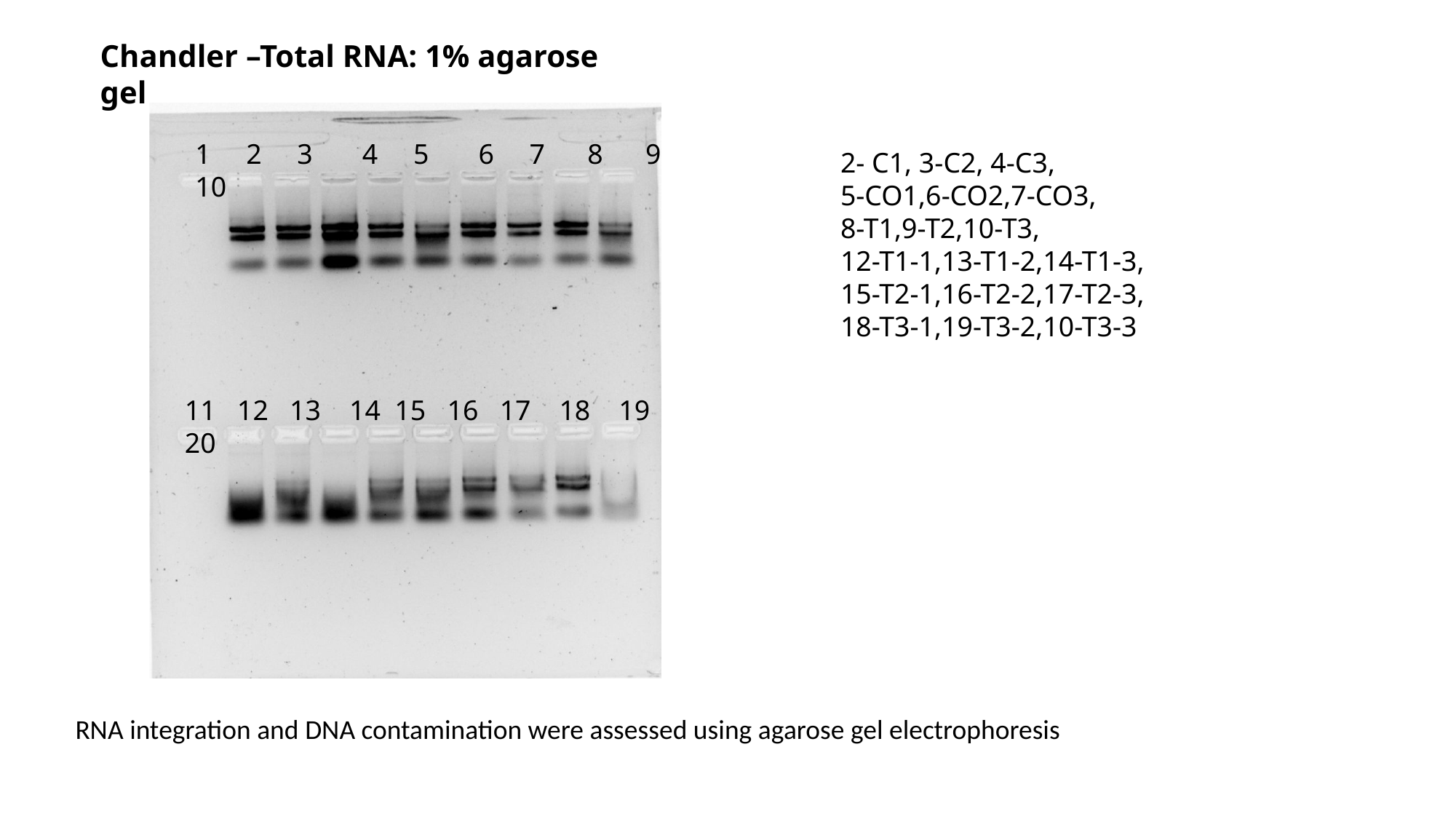

Chandler –Total RNA: 1% agarose gel
1 2 3 4 5 6 7 8 9 10
2- C1, 3-C2, 4-C3,
5-CO1,6-CO2,7-CO3,
8-T1,9-T2,10-T3,
12-T1-1,13-T1-2,14-T1-3,
15-T2-1,16-T2-2,17-T2-3,
18-T3-1,19-T3-2,10-T3-3
 11 12 13 14 15 16 17 18 19 20
RNA integration and DNA contamination were assessed using agarose gel electrophoresis

## Slide 2
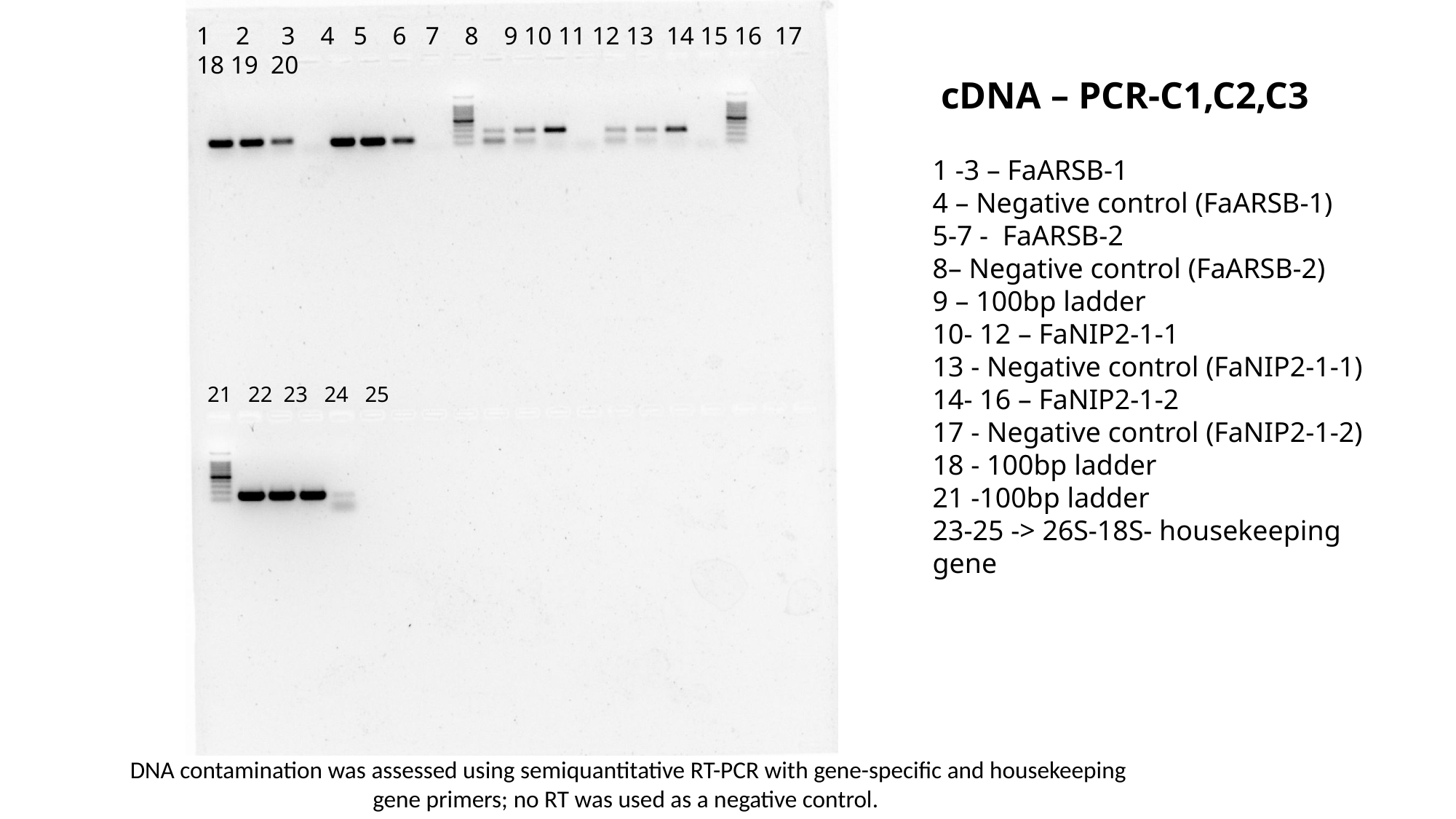

1 2 3 4 5 6 7 8 9 10 11 12 13 14 15 16 17 18 19 20
 21 22 23 24 25
cDNA – PCR-C1,C2,C3
1 -3 – FaARSB-1
4 – Negative control (FaARSB-1)
5-7 - FaARSB-2
8– Negative control (FaARSB-2)
9 – 100bp ladder
10- 12 – FaNIP2-1-1
13 - Negative control (FaNIP2-1-1)
14- 16 – FaNIP2-1-2
17 - Negative control (FaNIP2-1-2)
18 - 100bp ladder
21 -100bp ladder
23-25 -> 26S-18S- housekeeping gene
DNA contamination was assessed using semiquantitative RT-PCR with gene-specific and housekeeping gene primers; no RT was used as a negative control.
