# Supplementary material for: Investigating potassium silicate efficacy and mechanisms for improving the strawberry agronomic traits and gray mold fungal resistance
Source: PeerJ. 2026 Apr 29;14:e21151. doi: 10.7717/peerj.21151 (PMC13135329; doi:10.7717/peerj.21151)
Supplement: Supplemental Information 8 [file peerj-14-21151-s008.zip › Raw data/Rcode_with stats.docx]

library(agricolae)

DAS<- read.table("Yield.txt", header = T)

MKY<- aov(MKY ~ Treatment*Geno, data = DAS)

NMY<- aov(NMY ~ Treatment*Geno, data = DAS)

MN_MKY<-LSD.test(MKY, c("Treatment", "Geno"), group = TRUE, alpha = 0.05)

MN_NMY<-LSD.test(NMY, c("Treatment", "Geno"), group = TRUE, alpha = 0.05)

summary(MKY)

MN_MKY

summary(NMY)

MN_NMY

library(agricolae)

DAS<- read.table("Plantgrowth.txt", header = T)

HT<- aov(HT ~ Treatment*Geno, data = DAS)

Width<- aov(Width ~ Treatment*Geno, data = DAS)

MN_HT<-LSD.test(HT, c("Treatment", "Geno"), group = TRUE, alpha = 0.05)

MN_Width<-LSD.test(Width, c("Treatment", "Geno"), group = TRUE, alpha = 0.05)

summary(HT)

MN_HT

summary(Width)

MN_Width

library(agricolae)

DAS<- read.table("SPEC.txt", header = T)

ROOT<- aov(ROOT ~ TRT, data = DAS)

OCT<- aov(OCT ~ TRT, data = DAS)

NOV<- aov(NOV ~ TRT, data = DAS)

MN_ROOT<-LSD.test(ROOT, c("TRT"), group = TRUE, alpha = 0.05)

MN_OCT<-LSD.test(OCT, c("TRT"), group = TRUE, alpha = 0.05)

MN_NOV<-LSD.test(NOV, c("TRT"), group = TRUE, alpha = 0.05)

summary(NOV)

MN_NOV

summary(OCT)

MN_OCT

summary(ROOT)

library(agricolae)

DAS<- read.table("FUNGAL.txt", header = T)

ONE<- aov(ONE ~ TRT, data = DAS)

TWO<- aov(TWO ~ TRT, data = DAS)

THREE<- aov(THREE ~ TRT, data = DAS)

FOUR<- aov(FOUR ~ TRT, data = DAS)

FIVE<- aov(FIVE ~ TRT, data = DAS)

MN_ONE<-LSD.test(ONE, c("TRT"), group = TRUE, alpha = 0.05)

MN_TWO<-LSD.test(TWO, c("TRT"), group = TRUE, alpha = 0.05)

MN_THREE<-LSD.test(THREE, c("TRT"), group = TRUE, alpha = 0.05)

MN_FOUR<-LSD.test(FOUR, c("TRT"), group = TRUE, alpha = 0.05)

MN_FIVE<-LSD.test(FIVE, c("TRT"), group = TRUE, alpha = 0.05)

summary(ONE)

MN_ONE

summary(TWO)

MN_TWO

summary(THREE)

MN_THREE

summary(FOUR)

MN_FOUR

summary(FIVE)

MN_FIVE

getwd()

[1] "C:/Users/Amaranatha Vennapusa/Desktop/DSU/Rosalyn"

> library(agricolae)

> DAS<- read.table("SPEC.txt", header = T)

> ROOT<- aov(ROOT ~ TRT, data = DAS)

> OCT<- aov(OCT ~ TRT, data = DAS)

> NOV<- aov(NOV ~ TRT, data = DAS)

> MN_ROOT<-LSD.test(ROOT, c("TRT"), group = TRUE, alpha = 0.05)

> MN_OCT<-LSD.test(OCT, c("TRT"), group = TRUE, alpha = 0.05)

> MN_NOV<-LSD.test(NOV, c("TRT"), group = TRUE, alpha = 0.05)

> summary(NOV)

Df Sum Sq Mean Sq F value Pr(>F)

TRT 3 567.4 189.13 27.96 0.000136 ***

Residuals 8 54.1 6.76

---

Signif. codes: 0 ‘***’ 0.001 ‘**’ 0.01 ‘*’ 0.05 ‘.’ 0.1 ‘ ’ 1

> MN_NOV

$statistics

MSerror Df Mean CV t.value LSD

6.763029 8 33.28356 7.813413 2.306004 4.896492

$parameters

test p.ajusted name.t ntr alpha

Fisher-LSD none TRT 4 0.05

$means

NOV std r LCL UCL Min Max Q25 Q50

0mL 23.02309 3.467583 3 19.56075 26.48543 21.02108 27.02711 21.02108 21.02108

2mL 40.04016 1.733789 3 36.57782 43.50251 39.03916 42.04217 39.03916 39.03916

3mL 31.03184 1.733165 3 27.56950 34.49419 30.03120 33.03313 30.03120 30.03120

4mL 39.03916 3.003015 3 35.57681 42.50150 36.03614 42.04217 37.53765 39.03916

Q75

0mL 24.02410

2mL 40.54067

3mL 31.53216

4mL 40.54067

$comparison

NULL

$groups

NOV groups

2mL 40.04016 a

4mL 39.03916 a

3mL 31.03184 b

0mL 23.02309 c

attr(,"class")

[1] "group"

> summary(OCT)

Df Sum Sq Mean Sq F value Pr(>F)

TRT 3 1661.6 553.9 33.5 7.06e-05 ***

Residuals 8 132.3 16.5

---

Signif. codes: 0 ‘***’ 0.001 ‘**’ 0.01 ‘*’ 0.05 ‘.’ 0.1 ‘ ’ 1

> MN_OCT

$statistics

MSerror Df Mean CV t.value LSD

16.53315 8 40.29041 10.09197 2.306004 7.65583

$parameters

test p.ajusted name.t ntr alpha

Fisher-LSD none TRT 4 0.05

$means

OCT std r LCL UCL Min Max Q25 Q50

0mL 24.02410 3.003015 3 18.61061 29.43759 21.02108 27.02711 22.52259 24.02410

2mL 56.05622 6.251268 3 50.64273 61.46971 51.05120 63.06325 52.55271 54.05422

3mL 36.03614 3.003015 3 30.62265 41.44963 33.03313 39.03916 34.53464 36.03614

4mL 45.04518 3.003010 3 39.63169 50.45867 42.04217 48.04819 43.54368 45.04518

Q75

0mL 25.52560

2mL 58.55873

3mL 37.53765

4mL 46.54668

$comparison

NULL

$groups

OCT groups

2mL 56.05622 a

4mL 45.04518 b

3mL 36.03614 c

0mL 24.02410 d

attr(,"class")

[1] "group"

> summary(ROOT)

Df Sum Sq Mean Sq F value Pr(>F)

TRT 3 8568 2855.9 8.598 0.00696 **

Residuals 8 2657 332.2

---

Signif. codes: 0 ‘***’ 0.001 ‘**’ 0.01 ‘*’ 0.05 ‘.’ 0.1 ‘ ’ 1

> MN_ROOT

$statistics

MSerror Df Mean CV t.value LSD

332.1675 8 156.995 11.60895 2.306004 34.31571

$parameters

test p.ajusted name.t ntr alpha

Fisher-LSD none TRT 4 0.05

$means

ROOT std r LCL UCL Min Max Q25 Q50

0mL 153.5040 10.688074 3 129.2391 177.7689 144.1747 165.1657 147.6732 151.1717

2mL 125.1255 12.136532 3 100.8606 149.3904 111.1114 132.1325 121.6220 132.1325

3mL 150.1506 6.006025 3 125.8857 174.4155 144.1446 156.1566 147.1476 150.1506

4mL 199.1998 32.110236 3 174.9349 223.4647 171.1717 234.2349 181.6822 192.1928

Q75

0mL 158.1687

2mL 132.1325

3mL 153.1536

4mL 213.2139

$comparison

NULL

$groups

ROOT groups

4mL 199.1998 a

0mL 153.5040 b

3mL 150.1506 b

2mL 125.1255 b

[1] "group"

> library(agricolae)

> DAS<- read.table("FUNGAL.txt", header = T)

> ONE<- aov(ONE ~ TRT, data = DAS)

> TWO<- aov(TWO ~ TRT, data = DAS)

> THREE<- aov(THREE ~ TRT, data = DAS)

> FOUR<- aov(FOUR ~ TRT, data = DAS)

> FIVE<- aov(FIVE ~ TRT, data = DAS)

> MN_ONE<-LSD.test(ONE, c("TRT"), group = TRUE, alpha = 0.05)

> MN_TWO<-LSD.test(TWO, c("TRT"), group = TRUE, alpha = 0.05)

> MN_THREE<-LSD.test(THREE, c("TRT"), group = TRUE, alpha = 0.05)

> MN_FOUR<-LSD.test(FOUR, c("TRT"), group = TRUE, alpha = 0.05)

> MN_FIVE<-LSD.test(FIVE, c("TRT"), group = TRUE, alpha = 0.05)

> summary(ONE)

Df Sum Sq Mean Sq F value Pr(>F)

TRT 7 64.4 9.197 1.173 0.337

Residuals 46 360.7 7.841

> MN_ONE

$statistics

MSerror Df Mean CV

7.8406 46 27.862 10.04991

$parameters

test p.ajusted name.t ntr alpha

Fisher-LSD none TRT 8 0.05

$means

ONE std r LCL UCL Min Max Q25 Q50

0mL 27.20576 1.097233 9 25.32698 29.08453 25.98490 28.90658 26.40901 27.21012

1ml 30.37658 5.196732 3 27.12245 33.63072 26.88025 36.34823 27.39076 27.90127

1mL 28.00534 3.057496 6 25.70432 30.30635 24.24131 31.36880 25.48126 28.24488

2mL 27.06658 3.538959 9 25.18781 28.94536 21.29812 31.71830 25.51366 26.54646

3ml 24.89843 2.071250 3 21.64430 28.15256 22.90221 27.03733 23.82898 24.75575

3mL 28.87974 1.297887 6 26.57873 31.18076 27.05697 30.83866 28.28219 28.80055

4mL 28.21150 3.614785 9 26.33273 30.09028 23.77008 35.83379 26.45221 27.53606

5mL 28.33978 1.889207 9 26.46101 30.21856 25.71394 31.95785 26.97450 28.13689

Q75

0mL 27.41040

1ml 32.12475

1mL 30.59028

2mL 29.75481

3ml 25.89654

3mL 29.45439

4mL 28.47068

5mL 29.70769

$comparison

NULL

$groups

ONE groups

1ml 30.37658 a

3mL 28.87974 ab

5mL 28.33978 ab

4mL 28.21150 ab

1mL 28.00534 ab

0mL 27.20576 ab

2mL 27.06658 ab

3ml 24.89843 b

attr(,"class")

[1] "group"

> summary(TWO)

Df Sum Sq Mean Sq F value Pr(>F)

TRT 7 4229 604.2 5.45 0.000133 ***

Residuals 46 5100 110.9

---

Signif. codes: 0 ‘***’ 0.001 ‘**’ 0.01 ‘*’ 0.05 ‘.’ 0.1 ‘ ’ 1

> MN_TWO

$statistics

MSerror Df Mean CV

110.8656 46 58.56328 17.97931

$parameters

test p.ajusted name.t ntr alpha

Fisher-LSD none TRT 8 0.05

$means

TWO std r LCL UCL Min Max Q25 Q50

0mL 65.26222 9.062090 9 58.19745 72.32700 52.60990 77.77405 60.82123 68.71449

1ml 65.42367 14.945045 3 53.18711 77.66022 56.41122 82.67494 56.79803 57.18484

1mL 52.78331 7.970163 6 44.13076 61.43586 43.20475 64.34374 47.55923 51.30315

2mL 42.34869 2.428031 9 35.28392 49.41347 37.46349 45.91454 41.28445 42.41769

3ml 58.27654 3.414267 3 46.03999 70.51310 56.09706 62.21139 56.30912 56.52118

3mL 53.58641 11.362985 6 44.93386 62.23896 42.66676 68.82051 45.52462 49.07560

4mL 64.67684 11.663774 9 57.61206 71.74162 53.13799 88.74999 55.62582 58.73396

5mL 66.94535 15.305670 9 59.88057 74.01013 51.13335 90.73312 54.13750 62.96144

Q75

0mL 69.59413

1ml 69.92989

1mL 58.00114

2mL 42.88495

3ml 59.36628

3mL 62.92316

4mL 69.39778

5mL 82.56498

$comparison

NULL

$groups

TWO groups

5mL 66.94535 a

1ml 65.42367 ab

0mL 65.26222 ab

4mL 64.67684 ab

3ml 58.27654 ab

3mL 53.58641 b

1mL 52.78331 bc

2mL 42.34869 c

attr(,"class")

[1] "group"

> summary(THREE)

Df Sum Sq Mean Sq F value Pr(>F)

TRT 7 1064397 152057 6.184 3.97e-05 ***

Residuals 46 1131021 24587

---

Signif. codes: 0 ‘***’ 0.001 ‘**’ 0.01 ‘*’ 0.05 ‘.’ 0.1 ‘ ’ 1

> MN_THREE

$statistics

MSerror Df Mean CV

24587.41 46 679.2861 23.08361

$parameters

test p.ajusted name.t ntr alpha

Fisher-LSD none TRT 8 0.05

$means

THREE std r LCL UCL Min Max Q25

0mL 667.6465 111.11754 9 562.4366 772.8563 479.7997 779.8140 688.2051

1ml 684.5164 46.14792 3 502.2876 866.7452 633.5414 723.4499 665.0496

1mL 657.2729 147.88768 6 528.4177 786.1281 485.3329 837.2541 555.7173

2mL 437.1113 43.34411 9 331.9015 542.3211 337.7693 476.8570 436.1368

3ml 572.2034 43.19370 3 389.9746 754.4321 535.0525 619.5981 548.5060

3mL 736.0601 85.82449 6 607.2049 864.9153 593.9574 830.2130 712.8588

4mL 716.2541 183.44886 9 611.0443 821.4640 500.8366 962.2384 579.1493

5mL 906.9093 272.44553 9 801.6995 1012.1191 649.1826 1422.0144 701.2388

Q50 Q75

0mL 699.9036 714.2764

1ml 696.5579 710.0039

1mL 625.9211 784.1494

2mL 449.2769 462.5053

3ml 561.9594 590.7788

3mL 731.3549 799.9290

4mL 718.8474 850.9082

5mL 830.9041 1146.5871

$comparison

NULL

$groups

THREE groups

5mL 906.9093 a

3mL 736.0601 b

4mL 716.2541 b

1ml 684.5164 b

0mL 667.6465 b

1mL 657.2729 b

3ml 572.2034 bc

2mL 437.1113 c

attr(,"class")

[1] "group"

> summary(FOUR)

Df Sum Sq Mean Sq F value Pr(>F)

TRT 7 11466092 1638013 16.84 8.54e-11 ***

Residuals 46 4474550 97273

---

Signif. codes: 0 ‘***’ 0.001 ‘**’ 0.01 ‘*’ 0.05 ‘.’ 0.1 ‘ ’ 1

> MN_FOUR

$statistics

MSerror Df Mean CV

97272.83 46 2605.775 11.96903

$parameters

test p.ajusted name.t ntr alpha

Fisher-LSD none TRT 8 0.05

$means

FOUR std r LCL UCL Min Max Q25 Q50

0mL 2721.400 290.08957 9 2512.136 2930.665 2099.283 3033.699 2715.467 2808.623

1ml 2652.109 87.37788 3 2289.652 3014.566 2578.698 2748.756 2603.785 2628.873

1mL 2543.291 178.93846 6 2286.995 2799.586 2343.408 2772.935 2397.803 2525.350

2mL 1666.930 369.72278 9 1457.665 1876.194 1328.552 2375.837 1348.093 1517.173

3ml 2683.240 227.59017 3 2320.783 3045.697 2455.650 2910.831 2569.444 2683.238

3mL 2634.686 171.33903 6 2378.390 2890.982 2463.511 2925.302 2518.526 2588.880

4mL 2850.056 204.96788 9 2640.791 3059.321 2518.615 3107.483 2795.273 2880.075

5mL 3165.830 493.19569 9 2956.565 3375.094 2683.254 4191.124 2896.848 2933.384

Q75

0mL 2865.384

1ml 2688.815

1mL 2684.399

2mL 1940.036

3ml 2797.034

3mL 2707.071

4mL 2985.585

5mL 3501.796

$comparison

NULL

$groups

FOUR groups

5mL 3165.830 a

4mL 2850.056 b

0mL 2721.400 b

3ml 2683.240 b

1ml 2652.109 b

3mL 2634.686 b

1mL 2543.291 b

2mL 1666.930 c

attr(,"class")

[1] "group"

> summary(FIVE)

Df Sum Sq Mean Sq F value Pr(>F)

TRT 7 7191498 1027357 21.54 1.48e-12 ***

Residuals 46 2194366 47704

---

Signif. codes: 0 ‘***’ 0.001 ‘**’ 0.01 ‘*’ 0.05 ‘.’ 0.1 ‘ ’ 1

> MN_FIVE

$statistics

MSerror Df Mean CV

47703.61 46 5017.967 4.352591

$parameters

test p.ajusted name.t ntr alpha

Fisher-LSD none TRT 8 0.05

$means

FIVE std r LCL UCL Min Max Q25 Q50

0mL 5165.769 191.8661 9 5019.223 5312.316 4989.045 5581.550 4995.513 5167.496

1ml 5226.676 124.5248 3 4972.849 5480.502 5083.352 5308.349 5185.839 5288.325

1mL 5120.416 148.7566 6 4940.934 5299.899 4902.377 5294.691 5023.788 5150.219

2mL 4263.497 156.7948 9 4116.950 4410.044 4033.165 4512.615 4159.693 4248.741

3ml 5090.824 463.7392 3 4836.998 5344.650 4567.267 5449.917 4911.277 5255.288

3mL 5102.349 369.6160 6 4922.867 5281.831 4649.062 5541.769 4798.912 5128.371

4mL 4983.427 192.8339 9 4836.881 5129.974 4741.676 5423.685 4864.638 4965.168

5mL 5440.765 137.3251 9 5294.219 5587.312 5134.372 5621.346 5410.891 5424.290

Q75

0mL 5268.215

1ml 5298.337

1mL 5220.066

2mL 4337.007

3ml 5352.602

3mL 5390.163

4mL 5045.575

5mL 5509.541

$comparison

NULL

$groups

FIVE groups

5mL 5440.765 a

1ml 5226.676 ab

0mL 5165.769 b

1mL 5120.416 b

3mL 5102.349 b

3ml 5090.824 b

4mL 4983.427 b

2mL 4263.497 c

attr(,"class")

[1] "group"

libs = c('tidyverse','sommer','knitr','tidymodels','dplyr','pbkrtest','emmeans','car','ggplot2','readxl','agricolae', 'multcomp', 'multcompView','lme4','lmerTest')

library(tidyverse)

library(cowplot)

library(grid)

library(sommer)

library(ggplot2)

library(broom)

library(readxl)

library(agricolae)

library(lme4)

library(readxl)

library(ggplot2)

library(scales)

#####MBR Leaves

MBR_Leaves <- read_excel("~/Desktop/All folders/Rosayln Work/Graphs/Spec-silica-1 copy.xlsx")

MBR_Leaves$TRT=as.factor(MBR_Leaves$TRT)

ggplot(MBR_Leaves, aes(x = TRT, y = Leaves)) +

geom_bar(stat = "identity", position = "dodge", width = 0.5, fill = "#9ACD32") +

geom_errorbar(aes(ymin = Leaves - SE, ymax = Leaves + SE),

width = 0.4, position = position_dodge(0.7)) +

labs(x = "Potassium silica treatment", y = "Silica concentration (mol/L)", title = "Leaves") +

theme_minimal() +

theme(

axis.text.x = element_text(angle = 0, hjust = 1, face = "bold", size = 12),

axis.text.y = element_text(face = "bold", size = 12),

axis.title = element_text(face = "bold", size = 14),

legend.title = element_text(face = "bold", size = 12),

legend.text = element_text(face = "bold", size = 12),

plot.title = element_text(face = "bold", hjust = 0.5, size = 16),

panel.grid.major.x = element_blank(),

panel.grid.minor.x = element_blank(),

panel.grid.major.y = element_line(color = "gray70", linetype = "dashed")

) +

scale_fill_manual(values = "#9ACD32") +

coord_cartesian(ylim = c(0, max(MBR_Leaves$Leaves) * 1.1)) +

guides(fill = guide_legend(title = "Month"))

#####MBR Root

MBR_Root <- read_excel("~/Desktop/All folders/Rosayln Work/Ven/MBR Root.xlsx")

MBR_Root$Treatment=as.factor(MBR_Root$Treatment)

ggplot(MBR_Root, aes(x = Treatment, y = MBR)) +

geom_bar(stat = "identity", position = "dodge", width = 0.5, fill = "#CD853F") +

geom_errorbar(aes(ymin = MBR - SE, ymax = MBR + SE),

width = 0.4, position = position_dodge(0.7)) +

labs(x = "Potassium silica treatment", y = "Silica concentration (mol/L)", title = "Roots") +

theme_minimal() +

theme(

axis.text.x = element_text(angle = 0, hjust = 1, face = "bold"),

axis.text.y = element_text(face = "bold"),

axis.title = element_text(face = "bold"),

legend.title = element_text(face = "bold"),

legend.text = element_text(face = "bold"),

plot.title = element_text(face = "bold", hjust = 0.5),

panel.grid.major.x = element_blank(),

panel.grid.minor.x = element_blank(),

panel.grid.major.y = element_line(color = "gray70", linetype = "dashed")

) +

scale_fill_manual(values = "#CD853F") +

coord_cartesian(ylim = c(0, max(MBR_Root$MBR) * 1.1)) +

guides(fill = guide_legend(title = "Month"))

####### Trend graph

Trend <- read_excel("~/Desktop/All folders/Rosayln Work/Ven/Trend.xlsx")

Trend$Treatment=as.factor(Trend$Treatment)

# Define a custom color palette

custom_palette <- c("#E41A1C", "#377EB8", "#4DAF4A", "#984EA3", "#FF7F00")

# Define a custom theme

custom_theme <- theme_minimal() +

theme(

text = element_text(size = 14, color = "black"),

plot.title = element_text(size = 16, face = "bold", hjust = 0.5),

axis.title = element_text(size = 14, face = "bold"),

axis.text = element_text(size = 12),

legend.position = "right",

legend.title = element_text(size = 14, face = "bold"),

legend.text = element_text(size = 12),

panel.grid.major = element_line(color = "#F0F0F0"),

panel.grid.minor = element_blank(),

panel.background = element_rect(fill = "#FFFFFF")

)

# Create the plot

ggplot(Trend, aes(x = Treatment)) +

geom_point(aes(y = `1 DAI`, color = "Group 1"), size = 3) +

geom_line(aes(y = `1 DAI`, group = 1, color = "Group 1"), size = 1, linetype = "solid") +

geom_smooth(aes(y = `1 DAI`), method = "lm", se = FALSE, color = custom_palette[1], linetype = "dashed") +

geom_point(aes(y = `2 DAI`, color = "Group 2"), size = 3) +

geom_line(aes(y = `2 DAI`, group = 1, color = "Group 2"), size = 1, linetype = "solid") +

geom_smooth(aes(y = `2 DAI`), method = "lm", se = FALSE, color = custom_palette[2], linetype = "dashed") +

geom_point(aes(y = `3 DAI`, color = "Group 3"), size = 3) +

geom_line(aes(y = `3 DAI`, group = 1, color = "Group 3"), size = 1, linetype = "solid") +

geom_smooth(aes(y = `3 DAI`), method = "lm", se = FALSE, color = custom_palette[3], linetype = "dashed") +

geom_point(aes(y = `4 DAI`, color = "Group 4"), size = 3) +

geom_line(aes(y = `4 DAI`, group = 1, color = "Group 4"), size = 1, linetype = "solid") +

geom_smooth(aes(y = `4 DAI`), method = "lm", se = FALSE, color = custom_palette[4], linetype = "dashed") +

geom_point(aes(y = `5 DAI`, color = "Group 5"), size = 3) +

geom_line(aes(y = `5 DAI`, group = 1, color = "Group 5"), size = 1, linetype = "solid") +

geom_smooth(aes(y = `5 DAI`), method = "lm", se = FALSE, color = custom_palette[5], linetype = "dashed") +

labs(x = "Treatment", y = "Area (mm)", color = "Group") +

scale_x_discrete(labels = c("0 mL", "1 mL", "2 mL", "3 mL", "4 mL", "5 mL")) +

scale_color_manual(

values = custom_palette,

labels = c("1 DAI", "2 DAI", "3 DAI", "4 DAI", "5 DAI")

) +

scale_y_continuous(

breaks = seq(0, max(Trend$`5 DAI`), 500),

labels = comma

) +

custom_theme

library(ggplot2)

library(readxl)

library(doebioresearch)

data <- read_excel("C:/DSU_OneDrive/Strawberry/silicon transporters/Gene-expression-1-3-2025-R.xlsx",

sheet = "Sheet3")

#LSD and Duncan

LSD=crd(data[4],data$Treat,1)

LSD

DUNCAN=crd(data[4],data$Treat,2)

DUNCAN

sink("NIP2-1.txt")

print(LSD)

print(DUNCAN)

sink()

#Plots

rdata <- read_excel("C:/DSU_OneDrive/Strawberry/silicon transporters/Gene-expression-1-3-2025-R.xlsx",

sheet = "Sheet2")

View(rdata)

print(rdata)

rdata$Treatment <- factor(rdata$Treatment, levels = unique(rdata$Treatment))

ggplot(rdata, aes(x = factor(Treatment), y = NIP2, fill = Treatment, colour = Treatment)) +

geom_bar(stat = "identity", position = "dodge", alpha = 0.5) +

geom_errorbar(aes(ymin=NIP2-seNIP2, ymax=NIP2+seNIP2), position = position_dodge(0.85), width = 0.35, show.legend = FALSE, color = "black") +

labs(x="Treatment", y= "Relative Expression", title = expression(paste(italic("NIP2-1"), " Gene Expression")))+

theme_classic() +

theme(panel.grid.major = element_blank(), panel.grid.minor = element_blank()) +

geom_text(aes(label=DNIP2,

y = NIP2 + seNIP2 + 2),

position = position_dodge(0.9), vjust = 0.5, size = 4, colour = "black") +

theme(axis.text=element_text(size=12),

axis.title=element_text(size=14))+

scale_y_continuous(limits=c(0,25),expand = c(0,0))+

scale_fill_brewer(palette = "Dark2") +

scale_color_brewer(palette = "Dark2")+ theme(axis.text.x=element_text(colour = "black",size=12),axis.text = element_text(colour = "black", size=12))+

theme(legend.position = "none")+

theme(plot.title = element_text(hjust = 0.5))

ggsave("NIP2-1.png", width = 5, height = 4, dpi = 1000)
